# The uncertainty inherent to DEM simulations of interlocking particles

**DOI:** 10.1038/s41598-025-90129-6

**Published:** 2025-03-04

**Authors:** Lukas Maier, Michael Mitterlindner, Hadie Benabchiasli, Gregor Fasching, Stefan Radl

**Affiliations:** Institute of Process and Particle Engineering, Graz University of Technology, Inffeldgasse 13/III, 8010 Graz, Austria

**Keywords:** Discrete element method, Simulation, Non-spherical particles, Machine learning, Chemical engineering, Computational science

## Abstract

In industrial applications, the handling of heterogeneous mixtures of phases and materials poses challenges for direct measurements and experiments, necessitating complementary modeling approaches. The Discrete Element Method (DEM) is commonly used for simulating the flow of granular systems, typically with spherical particles. However, certain applications, such as recycled polymers and batteries, require alternative non-convex particle representations in DEM simulations. Tetrapods are a promising shape candidate for modeling the flow behavior of such materials, as well as the associated uncertainty. We investigate the impact of the tetrapods’ properties on the outcome and uncertainty inherent to DEM-based simulations. We demonstrate that tetrapods are effective for modeling interlocking materials, with their shape and size parameter significantly affecting interlocking behavior. Most interestingly, we can correlate the shape and size of tetrapods to the uncertainty inherent to our simulations. Specifically, we find that this uncertainty is positively correlated with both tetrapod size and the interlocking parameter *ξ/D* that quantifies their non-convexity. Lastly, we provide guidelines for selecting optimal tetrapod parameter sets for accurately modeling materials based on mean and variability measured in experiments.

## Introduction

### Motivation

In a real-world application, the variety of powders and particles to be found is immense. To use, transport, and store them appropriately, one has to know how such granular material actually behaves. For this purpose, an accurate approach to model real, rarely ideal, particles is necessary. Nonetheless, most research in this regard is focused on idealized, spherical particles. In this regard, the so-called Discrete Element Method (DEM) simulation plays an important role. This method enables researchers and engineers to investigate a system’s behavior based on the knowledge of individual particle properties (or that of parcels, which we will introduce later) by utilizing individual particle tracking.

The modeling of real granular systems, especially poorly flowing or interlocking ones like pulp, fibrous material, or glass wool is extremely challenging and typically cannot be achieved by using spherical particles^1–3^. One such case is the description of ground polymeric materials (“plastics”) or batteries (i.e., a “recyclate” that is then refined to recover critical raw materials). Industrially, recycled batteries are projected to gain relevancy due to the necessity to increase the recycling rate^4^. Currently, also only about 20% of plastics are being recycled, while it is necessary to increase this number to 75% to fulfill the requirements of a sustainable plastic lifecycle^5^. In this regard, battery recycling may play an important role in the near future. This recyclate stream often contains dangerous dust particles, tends to self-heat, and requires complex testing procedures (due to chemical reactions and evaporation of the moisture that is part of the electrolyte). Thus, experiments should be fast, simple, and should be reduced as much as possible.

**Fig. 1:**
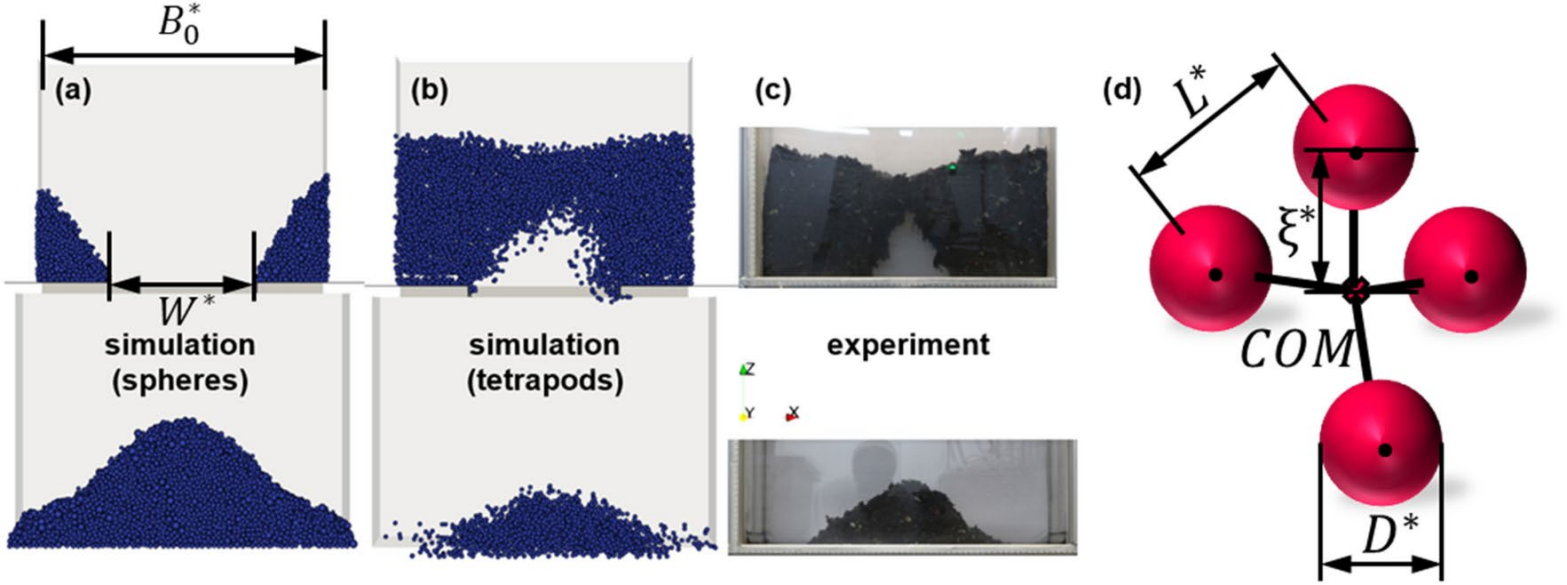
(**a**) Illustration of the simulation domain with the reference box width ${B}_{0}^{*}$, gap width ${W}^{*}$, and the region used for arching detection in red; results of calibrated simulations using (**a**) spheres and (**b**) tetrapods; (**c**) experimental results with recyclate; (**d**) sketch of a tetrapod illustrating the edge length ${L}^{*}$, the diameter of spheres ${D}^{*}$, the tetrapod radius ξ*, and the center of mass (COM) position (adapted from Mitterlindner et al.^43^ and Radl et al. ^8^).

To gain meaningful results from typical numerical simulations, one first needs to adjust certain model parameters using experimental data, the so-called calibration^6^. Our preliminary experiments using a commonly used testing device for calibration of DEM parameters (a drawdown tester introduced later), show strong interlocking and arching within this granular structure made up of recyclate material^7^. This behavior cannot be predicted when using a DEM simulation with spheres, even in cases considered extremely cohesive in our simulations. Thus, a concept first described by Radl et al.^8^, a so-called tetrapod-shaped parcel was adopted to model the behavior of strongly interlocking particles. “Model” in this context means that an ensemble of particles is abstracted to a geometrically simple object (e.g., a tetrapod) whose behavior should match the particle ensemble. In Fig. 1 the simulation using spheres (panel a), using tetrapods (panel b), the real experimental results with recyclate (panel c), and the concept of a tetrapod-shaped parcel (panel d) is illustrated. As can be seen, an ensemble of such tetrapod-shaped parcels (as indicated by our simulation results in Fig. 1b) can approximate real-world particles (i.e., Fig. 1c) reasonably well.

Unfortunately, and as will be shown in our present study, tetrapods introduce a high variability and dependence on the initial conditions into the DEM simulation. Mainly, this is due to their shape, which allows them to interlock in a plurality of configurations, depending on their relative position and orientation.

In most previous DEM studies, the uncertainty between individual simulation runs is not considered, or deemed to have negligible impact on the results of the simulation^9^. This approach seems dangerous in case the variability, or “rare events” are important for the usability of the results. As the application of non-spherical particles in DEM simulations is a rather new development^10^, the variability was not investigated in relevant prior studies to the best of our knowledge. As we show in our present work, the sensitivity to small variations in the initial parameters such as position, translational speed, rotation rate, or cohesion is low when using spherical particles. Additionally, the variability is often neglected, simply because DEM-based simulations are expensive. Especially, the issue of packing-related uncertainty, which is of special interest for extremely non-convex particles (a prime example being the tetrapods considered by us), is not covered by literature yet. We will carefully discuss this gap in knowledge in section "Detailed literature review" below.

Our interest in the quantification of the uncertainty of DEM-based simulations is fueled by two recent developments: Firstly, our own experimental results show a large variability, which—as we will show—cannot be predicted when using spherical particles. Especially, the issue of arching and non-arching cases being observed, and how this relates to the shape and size of tetrapods, is of central interest to us. Secondly, advances in computational power and the establishment of high-performance computing (HPC) allows a large number of DEM-based simulations to be performed. Thus, we are now able to investigate the statistics of such simulations in an exhaustive manner in a reasonable timeframe and a plurality of particle shapes.

### Detailed literature review

Firstly, we want to review the current state-of-the-art research concerned with DEM simulations focused on uncertainty prediction and calibration. Generally, the calibration of DEM simulation parameters is a well-studied topic as researchers constantly try to make the calibration procedure more accurate and efficient. However, there are many different aspects, some of which are already discussed in literature. For example, when it comes to calibration strategies for a wide range of particles, the article of Coetzee^11^ investigates a plurality of testers. One of these testers is the drawdown tester we use in our study. Their findings nicely support our choice of this specific testing device. However, the main topic studied in our work, the uncertainty, is not covered. In contrast, this aspect is investigated by Cheng et al.^9^ in their work on probabilistic calibration. However, their focus is exclusively on spherical particles, where they deem the grain configuration to have a negligible impact on the results of their simulations. Thus, they used a “prototype” packing for their analysis. This assumption, supported by our own preliminary simulations using spheres and tetrapods, definitely does not hold for non-convex particles such as tetrapods. Another approach to tackle uncertainty quantification was made by Dahl et al.^12^. They tried to narrow down the most important variables for each simulation case by applying the results of a prior sensitivity analysis to their data. Thus, they reduced the number of variables to be looked at in their extensive grid-search approach, and also narrowed the step-size used within this search. In our Supplementary Information (see section A.6), we looked at the applicability of their method to our set of problems. Interestingly, their approach does not seem to impact the results of our simulations in terms of uncertainty, but just shifts the (mean) expectancy $\overline{x }$. An extensive explanation of the method can be found in our Supplementary Information (see section A.6).

An early attempt to assess the viability of various particle and parcel approaches was made by Bierwisch et al.^13^. They investigated the impact of four different models: (i) rotating spheres, (ii) non-rotating spheres, (iii) overlapping multi-spheres, and (iv) non-overlapping multi-spheres on the properties of granular systems in cavity filling simulations. However, their analysis did not account for uncertainty in the results. An even earlier contribution was provided by Pöschel and Buchholtz^14^, who explored the behavior of static friction in granular materials using a 2D system with non-spherical particles. Their findings indicated that the approach could serve as a viable description of the phenomenon. Nevertheless, their study was limited to single simulations and confined to two dimensions, without considering uncertainty. Both studies neglected to account for the potential role of “interlocking” among parcels or non-spherical particles in influencing the observed effects. This omission could be a critical factor in understanding the underlying mechanics of such systems.

In summary, none of the above publications dealt with non-spherical particles and considered the associated variability in a systematic manner. Thus, we next summarize relevant literature in the field of non-spherical particles that helps us to conclude on the expected uncertainty of such simulations.

As already mentioned, preliminary testing in our study confirmed that the initial packing has a large impact on simulation results involving tetrapods. This is an issue considered by Rolland et al.^15^ in their paper on predicting the average void fraction and the associated uncertainty. Specifically, they considered non-spherical particles, so-called “poly-lobed” particles. They established that random input parameters (such as location and orientation) have a considerable impact on the outcome of the simulation experiments, which supports our hypothesis. However, the number of simulations done in this prior study was very limited. The effect of such a small number of simulations is discussed in section "Parcel Size Effect", where we compare the data gained in our present study to the data published by Rolland et al.^15^.

When it comes to the modeling of interlocking phenomena in particulate systems, the work of Blair and Ness^16^ investigated micron-sized solids in dense suspensions. However, they considered a 2D domain and only approximately 100 particles having a simple interlocking shape (i.e., a sphere with small spherical asperities) without estimating the associated uncertainty. Datta et al.^17^ dealt with the influence of shape parameters of superquadric-modelled particles on the outcome of a DEM simulation. However, they did not consider the uncertainty associated with their predictions.

Finally, the most recent publication concerned with non-spherical, interlocking particles is that by Riedel et al.^18^. They dealt with cluster formation of microswimmers, a relevant topic in biological systems (e.g., for the formation of biofilms). In their work, they focused on the phenomenological description of the interlocking behavior of different shaped microswimmers in a transient sense. Furthermore, the simulation was done in 2D in a rather dilute system without considering the associated uncertainty.

### Gaps in knowledge

In summary, there is no conclusive picture about how the shape of particles impacts uncertainty inherent to experiments or 2D and 3D DEM simulations. This indicates that further research is needed to better understand the complex interplay between particle geometry and the outcome and accuracy of simulations. Furthermore, real-world particles are rarely homogeneous with regards to their shape and size. Thus, modeling of the actual particles and their individual property distributions is practically impossible, which brings up the need for alternative approaches. One such approach, parcels which are in itself an ensemble of particles, is used in our present work. Thus, it is important to note that the issue of using parcels to model shape effects is also not well understood. This is especially true when it comes to describing the uncertainty associated with using parcels to model real non-spherical particles^19,20^. Clearly, the knowledge of the effects of parcel properties, such as the shape and the size, on the uncertainty is extremely limited. This is especially true when it comes to interlocking parcels, which is a practically unstudied area of research. Aside from the work by Washino et al.^1^, which focused on coarse-graining DEM simulations using non-spherical and poly-dispersed particles, no substantial approaches have been made to address this issue. When it comes to transient simulations, such as the drawdown tester, there is – to the best of our knowledge—no information available about the uncertainty of important flow properties, such as the tendency of arching ${\upeta }_{arch}$ (we will define this parameter in section "General definitions").

Also, in all recent prior publications^11,12,21^, a calibration using non-spherical particles exclusively uses mean experimental data, not accounting for the deviations between runs. However, such particles may introduce considerable uncertainty due to their shape. Following this thought, one would anticipate a significant impact from these deviations, making the uncertainty a notable concern^22,23^.

Lastly, it is yet to be established how the concept of uncertainty is to be integrated into existing calibration procedures appropriately. For example, there is no publication in the context of the DEM that conclusively illustrates how fluctuations seen in experiments can be matched with DEM predictions.

### Goals

In a first approach, our present work shall demonstrate the need for alternative particle and parcel shapes, such as tetrapods. Specifically, the applications of interlocking parcels will be outlined.

Secondly, the influence of the parameters defining the shape of a tetrapod, such as the tetrapod radius ${\upxi }^{*}$ and the diameter of the spheres ${D}^{*}$, will be extensively studied. A special focus will be on the factors which influence the uncertainty in the predictions for the system behavior the most. Also, the overall uncertainty of specific simulation configurations will be investigated and conclusively discussed.

Finally, a potential strategy to find the optimal parcel parameters for a given mean and variability will be outlined and its applicability will be tested and validated. Using this information, one will be able to design tetrapods optimally in terms of their ability to reflect experimental data at minimal computational expense.

## Methods

### DEM

Discrete element method (DEM) simulation is a computational method used to model granular flows^24^. In contrast to computational fluid dynamics (CFD) simulations, which models the macroscopic flow behavior using an Eulerian approach, DEM tracks each particle within a given simulation domain individually (i.e., DEM uses a Lagrangian approach) and solves the corresponding momentum balances. Furthermore, it detects particle–particle and particle–wall binary collisions by overlap-detection, which is called “soft-sphere” approach^25^. In this regard, “soft-sphere” means, that the particles can overlap and collisions do not happen instantaneously, in contrast to the “hard-sphere” approach^26^. The collisions are traditionally modeled using a spring-dashpot model to account for inelastic collisions. In the following Eqs. (2-1)–(2-10) different specifics of said model are introduced.

What follows within section "DEM" is, if not stated otherwise, adapted from Radl^27^.

Generally, DEM simulations with a “soft-sphere” approach solve the differential form of Newton’s 2nd law of motion as stated in Eq. (2-1) for translational, and in Eq. (2-2) for rotational motion. They describe the differential rate of change of translatory and rotational momentum for a particle *i*.

2-1 $$\frac{d\left({{m}_{i}}^{*}{{{\varvec{v}}}_{i}}^{*}\right)}{d{t}^{*}}={{m}_{i}}^{*}\frac{d{{{\varvec{v}}}_{i}}^{*}}{d{t}^{*}}={{m}_{i}}^{*}\frac{{d}^{2}{{{\varvec{x}}}_{i}}^{*}}{d{{t}^{*}}^{2}}= \sum {{{\varvec{f}}}_{i}}^{*}$$

2-2 $$\frac{d\left({{\mathbf{I}}_{i}}^{*}{{{\varvec{\upomega}}}_{i}}^{*}\right)}{d{t}^{*}}=\sum {{\mathbf{t}}_{i}}^{*}$$

As said, the DEM simulation with “soft-spheres” relies on collisions for particles to exert forces onto each other. These forces can be split up into contact forces (${{{\varvec{f}}}_{C}}^{*}$), non-contact forces (${{{\varvec{f}}}_{NC}}^{*}$), and fluid forces (${{{\varvec{f}}}_{f}}^{*}$), in addition to gravitational forces. The sum of forces is defined in Eq. (2-3).

2-3 $${{{\varvec{f}}}_{i}}^{*}=\sum {{{\varvec{f}}}_{C}}^{*}+{{m}_{i}}^{*}{{\varvec{g}}}^{\boldsymbol{*}}+\sum {{{\varvec{f}}}_{NC}}^{*}+\sum {{{\varvec{f}}}_{f}}^{*}$$

Similarly, the torques can be split up into contact torques (${{{\varvec{f}}}_{C}}^{\boldsymbol{*}}\times {{{\varvec{r}}}_{C}}^{*}$) and other torques (${{{\varvec{t}}}_{*}}^{*}$), as shown in Eq. (2-4).

2-4 $${{{\varvec{t}}}_{i}}^{*}=\sum {{{\varvec{f}}}_{C}}^{\boldsymbol{*}}\times {{{\varvec{r}}}_{C}}^{*}+{{{\varvec{t}}}_{*}}^{*}$$

The contact forces are, as shown in Eq. (2-5), composed of the normal force (${{{\varvec{f}}}_{n}}^{*}$) and the tangential force (${{{\varvec{f}}}_{t}}^{*}$), which causes translational and rotational motion changes.

2-5 $${{{\varvec{f}}}_{C}}^{*}={{{\varvec{f}}}_{n}}^{*}+{{{\varvec{f}}}_{t}}^{*}$$

#### Force model

The normal forces exerted on particle $i$ can be described as the sum of the elastic (${{{\varvec{f}}}_{n}^{el}}^{*}$) and the viscous (${{{\varvec{f}}}_{n}^{vis}}^{*}$) normal forces, multiplied with the normal vector $\widehat{{\varvec{n}}}$. In common DEM simulation software, such as LIGGGHTS® used in our present work, the normal forces are defined as always being repulsive, hence the bounding with 0 to prevent negative values as shown in Eq. (2-6). This is important as otherwise, attractive forces would be possible only due to contact forces (e.g., for ${{f}_{\text{n}}^{\text{vis}}}^{*}\ll 0$), which is physically less meaningful.

2-6 $${{{\varvec{f}}}_{n,i}}^{*}=max\left({{{\varvec{f}}}_{n}^{el}}^{*}+{{{\varvec{f}}}_{n}^{vis}}^{*},0\right)\widehat{{\varvec{n}}}$$

The elastic force is used to model the conservation of energy during a binary collision and is always a function of the overlap ${\delta }^{*}$. Depending on the goal, linear or non-linear models can be used to describe the corresponding function. In our study, we used the Hertzian contact model, which is based on the theory of elasticity for smooth spheres. It uses a non-linear function in terms of ${\delta }^{*}$, as stated in Eq. (2-7). The coefficient ${k}_{hz}$ is a function of the effective Youngs modulus ${{Y}_{eff}}^{*}$ and the effective radius ${{r}_{eff}}^{*}$, both given by the properties of a granular material.

2-7 $${{{\varvec{f}}}_{n}^{el}}^{*}={k}_{hz}{{{\delta }_{n}}^{*}}^{3/2}$$

$${k}_{hz}=\frac{4}{3}{{Y}_{eff}}^{*}\sqrt{{{r}_{eff}}^{*}}$$

For the viscous force on the other hand, which generally represents the amount of dissipated energy during collision, a number of different models exist. The one used in our work is based on Tsuji et al.^28^. The full documentation can be found in the LIGGGHTS®-PUBLIC Documentation, Version 3.X^29^. The more general definition of the viscous normal force is stated in Eq. (2-8), where it becomes clear why the normal force has to be bounded. ${{{\varvec{f}}}_{n}^{vis}}^{*}$ a function of ${\dot{{\delta }_{n}}}^{*}$*,* which represents a change of overlap in the normal direction. If the particles reduce their overlap, this term becomes negative and would, if the change happens sufficiently fast, cause the whole term to become negative, thus attractive.

2-8 $${{{\varvec{f}}}_{n}^{vis}}^{*}={{c}_{n}}^{*}{\dot{{\delta }_{n}}}^{*}$$

The tangential force can be split up using the same concept as the normal force, by using a spring-dashpot model. In this regard, the first term in Eq. (2-9) acts as elastic, spring term, while the second term is dissipative. To use this approach, one needs to know the value and rate of change of the instantaneous tangential overlap ${{\delta }_{t}}^{*}$, which is the reason, this method is called “history”-approach.

2-9 $${{F}_{t}}^{*}={\text{mi}}{\text{n}}\left({k}_{t}{{\delta }_{t}}^{*}+{{c}_{t}}^{*}{{\dot{\delta }}_{t}}^{*},\mu \left|{{{\varvec{f}}}_{n}}^{*}\right|\right)$$

Finally, many DEM simulations use some sort of cohesion models. One approach in this regard, already implemented in LIGGGHTS®, is called the simplified Johnson-Kendall-Roberts (sjkr) theory for soft particles. It acts into the same direction as ${{{\varvec{f}}}_{n}}^{*}$ and is defined in Eq. (2-10), where ${A}^{*}$ is the particle contact area and ${{k}_{sjkr}}^{*}$ the variable cohesion energy density^30,31^.

2-10 $${{{\varvec{f}}}_{coh}}^{*}={{k}_{sjkr}}^{*}\cdot {A}^{*}$$

The rolling torque is described in our present study with the alternative elastic–plastic spring-dashpot as defined by Iwashita and Oda^32^.

### Shape model

We focus on the possibly simplest way to describe non-sphericity: the (glued) multi-sphere approach (i.e., particle consists of multiple rigidly connected “atoms” to represent its shape). We will refer to these “atoms” as “spheres” in what follows. The classical multi-sphere approach is problematic in cases where (i) the shape cannot be well represented by spheres, or (ii) multiple spheres contribute to a single contact^33^. Both challenges are not relevant for us, as will be clear from the next section.

The classical multi-sphere approach uses (strongly) overlapping spheres, and has the aim to represent real-world particles. In contrast, tetrapods are non-overlapping multi-sphere parcels that abstract an ensemble of strongly interlocking particles.

Further details and applications of other models for (non-spherical) particle shape representations are discussed in Ma et al.^34^ and Lu et al.^10^. Such other shape models would typically require significantly more computational resources as the multi-sphere approach. Hence, they were not considered by us.

### Parcel approach

Traditionally, DEM simulations use spherical particles to model real ones, which—as one can imagine—rarely can be described as actually being spherical. Also, the large number of particles present in relevant systems poses a challenge. To deal with such cases, Radl et al.^20^ investigate the concept of using “parcels” of particles, i.e., a cloud of particles, in the context of the soft-sphere DEM. Such parcels are typically modeled as (i) (multi-)spheres, or (ii) superquadrics^1,8,13^. The former uses an arrangement of spheres which make up the full particle, thus this approach is able to describe non-spherical particles again by using spheres. The parcel used in the present work uses multi-spheres and is shown in Fig. 1 (d). On the other hand, the concept of superquadrics is based on using a 3D surface with an equation and five parameters to fully describe the shape of said surface. A first effort to apply these two concepts to non-spherical parcels was made by Washino et al.^1^. Another relevant work in this regard, applying parcels to problems in cohesive gas-particle flows is the one by Tausendschön et al.^35^, which used spheres though.

Our preliminary work shows that strong interlocking cannot be modelled efficiently with superquadrics, and that multi-spheres (with relatively small number of spheres) would be a promising approach. The former is due to the more complex contact detection of superquadrics, thus requiring more computational effort for the same number of particles or parcels^1^. More specifically, we want to use non-connected spheres within our multi-sphere to describe the behavior of granular flows with strong interlocking. Our tetrapods are parcels of tetrahedral outer shape, with the four shape-defining spheres being located at each of the vertices of said tetrahedron. The spheres are connected to the center of mass (COM) via virtual “rods”. The “rods” are similar to bonds in a molecular system. However, they are rather a mathematical concept than a real physical bond between the spheres and the COM. The rod length is fixed, and hence all spheres are located at the same distance from the COM. Clearly, the length of the rods (next to the size of the spheres) is an important interlocking parameter.

One may ask why tetrapods were chosen in the first place, and not other non-convex shapes. The answer to this question is twofold: (i) we chose tetrapods for their ability to interlock by shape. Indeed, tetrapods represent the simplest geometry with a three-dimensional base shape offering shape interlocking – all other objects with a polyhedral base shape are more complex. (ii) the base shape (i.e., a tetrahedra) is used in many other modelling approaches as well (e.g., tetrahedra-based meshes in the context of continuum models, an example are meshes used in the Finite Element Method).

### Test setup

The so-called calibration of a DEM simulation uses experimental data to adjust the parameters of the simulation to fit the experimental data optimally. In this regard, a number of different standardized tests exist, which are used to find characteristic features of a granular system. One such testing device is the drawdown tester, as proposed by Roessler et al.^7^. Building upon their work, the drawdown tester used in this work has an upper box with the dimensions of 495 × 100 × 400 mm (xyz) and a lower box with 580 × 400 × 180 mm (xyz). Although not relevant for the gained experimental results, the deviations from the original publication arise from limitations due to the size of the fume hood (which needs to be used in experiments with battery recyclate), which made a lower total height necessary.

The rectangular flap, basically a trapdoor connecting the upper and lower box, is set to a width ${W}^{*}$ of 250 mm. During testing, the granular material is filled into the upper box up to a height of 250 mm, the flap is opened, leading to a granular flow into the lower box. A pile forms in the latter, which has some important geometrical characteristics to be measured, and which can be used when calibrating a DEM-based model. In this case, the heap mass fraction ${\upomega }_{heap}$ and the arching fraction ${\upeta }_{arch}$ are the most important factors. The former can be measured gravimetrically. The classification into arched and non-arched cases is determined through visual observation of the formation and persistence of an arch during and after the drawdown test.

The simulation domain is constructed with the same geometry as the experimental setup. The simulation itself is run in a series of sequential steps: Firstly, the parcels are inserted into a domain higher than the target height of 250 mm. Specifically, the insertion region has double the height of the target bed height in the top box. This is due to the insertion algorithm used by LIGGGHTS® being unable to insert a sufficiently high particle volume fraction ${\upphi }_{p}$ (this is due to the collision detection that must be performed when inserting particles). Following this, the simulation is run until the parcels have settled in the upper box. The settling-criterion ${\uplambda }_{settle}$ is defined as the fraction of the total kinetic energy of all parcels ${{E}_{kin}}^{*}$ and the initial potential energy ${{E}_{pot}}^{*}$ of the system, as shown in Eq. (2-11). The threshold after which the settling is set to be complete is ${10}^{-7}$. ${{E}_{pot}}^{*}$ in this regard is calculated with the lower reference level being the bottom of the upper box and the upper limit of the insertion region being the upper reference level.

2-11 $${\uplambda }_{settle}=\frac{{{E}_{kin}}^{*}}{{{E}_{pot}}^{*}}={10}^{-7}$$

Upon reaching said threshold, all parcels which surpass the upper limit of the desired filling height (250 mm) are cut off, including parcels that only partly do so. This loop of settling and deleting parcels is repeated 2 times to improve repeatability. If the threshold is not reached, this part of the simulation terminates after $1\cdot {10}^{6}$ steps to prevent infinite runtime due to shortcomings inherent to our choice of torque models (see discussion below).

Following this, the flap is opened within 0.5 *s* by sliding it in the negative y-direction (similarly to what is done in our experiments). Subsequently, the parcels begin to fall through the, now-open, flap into the lower box and form a heap. The settling-criterion is defined in the same way as for the settling outlined in the previous subsection and stated in Eq. (2-11). The upper reference level is the top of the lower box and the lower one the bottom of it. By just using this criterion, very few (< 1%) simulations stay in an infinite loop due to the following shortcoming of the torque models used: (i) we do not use a twisting torque model (to keep the model simple), and (ii) we did not fine-tune our rolling torque model to prevent this behavior. To avoid such an infinite loop, we set an upper limit of time steps, in this case ${2}\cdot {10}^{6}$. This has no impact on the results of this < 1% of simulations, since only a few parcels (of at least 2000 parcels in such a simulation) show this behavior.

All other parameters used in this setup are shown in Table 1. The cohesion energy density between particles *k*_sjkr_^*^ is an important parameter in DEM simulations, which is typically adjusted during calibration. However, this is not the focus of this work, thus the results from the prior work by Radl et al.^8^ are used. More important, the cohesion energy density between particles and the wall is set to 0 to avoid particle–wall sticking that is absent in experiments. All other variables are taken from Ajmal et al.^21^ as they already investigated a similar test setup.

**Table 1:** Fixed DEM parameters used in all simulations taken from Ajmal et al.^21^ and Radl et al.^8^ .

Parameter (p = particle, w = wall)	Value	Unit
Young’s modulus Y* [p, w]	${10}^{8}$	[Pa]
Poisson ratio ν [p, w]	$0.30$	[-]
Coefficient of restitution *e* [pp, pw]	$0.30$	[-]
Sliding friction coefficient ${\mu }_{rs}$ [pp]	$0.71$	[-]
Sliding friction coefficient ${\mu }_{rs}$ [pw]	$0.41$	[-]
Rolling friction coefficient ${\mu }_{rr}$ [pp,pw]	$0.80$	[-]
Particle density ${{\uprho }_{p}}^{*}$ [p]	$2900$	[kg/m^3^]
Cohesion energy density k_sjkr_* [pp]	$\text{3.1}\cdot {10}^{5}$	[J/m^3^]
Time step ${t}^{*}$	$\text{0.5}\cdot {10}^{-5}$	[s]

All simulations are conducted on an HPC-cluster using 20 cores for a typical simulation. However, the parcel insertion is completed prior to the actual drawdown test, utilizing only one core. This approach is taken to avoid the influence of inter-processor boundaries when introducing new parcels into the simulation domain. For the drawdown phase, prior tests have shown that the number of cores has a negligible effect on the statistical results as it should be.

### Statistical considerations

The null-hypothesis for almost any randomly distributed data is, that the underlying distribution is normally distributed. To test this hypothesis, the gained DEM simulation data for the heap mass fraction ${\upomega }_{heap}$ (at ${N}_{S}$ = 31.3, $\upxi /D$ = 2.17, and for *n* = 980 individual simulations) is examined using the approach of Kolmogorov–Smirnov^36^. The corresponding number-based cumulative density function (CDF) is shown in the Supplementary Information (see Figure A-2). The threshold value for $\alpha$ = 0.05 is ${\upbeta }_{T}$ = 0.043 and is hence bigger than the observed value of $\upbeta$ = 0.032. Thus, the null-hypothesis is supported, and a normal distribution can be safely assumed^37^.

Under the assumption that the model output data is normally distributed, the empirical data can be estimated using student-t-distributions considering the sample size *n*, the mean $\overline{x }$, and the empirical standard deviation *s*. Furthermore, the necessary number of simulations to reach a certain confidence level for the bounds within which the true mean *µ* is located, can be calculated. This is an important piece of information when working on gaining useful information for further studies on the variability of results using non-spherical particles.

To be able to compare the different empirical distributions to one another, one has to use a normalized version of a variability measure. One such measure is the index of dispersion (*ID*) defined by the variance ${s}^{2}$ of a normal distributed variable normalized by its mean^38^. The corresponding mathematical definition is shown in Eq. (2-12).

2-12 $$ID=\frac{{s}^{2}}{\overline{x} }$$

The confidence interval at a two-sided confidence level of $1-\alpha$ and the width $\overline{x }\pm \left(\overline{x}\cdot \text{E }\right)$ can be found using Eq. (2-13). The value for $t$, based on the student-t-distribution, is tabulated and reaches 1.96 for a large enough sample count *n*^39^. In our case, each simulation was typically run 300 times (i.e., *n* = 300), hence $t=1.97.$

2-13 $$n={\left(\frac{{t}_{1-\frac{\alpha }{2},\hspace{0.17em}n-1}\cdot s}{E\cdot \overline{x} }\right)}^{2}$$

For all considerations in our present work, reaching a relative error *E* of 5% with a confidence level of 95% is the goal (i.e., $\alpha$ = 0.05, $E$ = 0.05). This ensures, that with 95% confidence, the real mean *µ* of our population with regard to the target variable (in this case ${\omega }_{heap}$) is within $\overline{x }\pm 5 \%$.

### General definitions

In the DEM simulations used in our present work, the starting configuration (after inserting particles into our simulation) strongly influences the results of the simulations. Slight variations in this insertion may lead to large deviations during the actual simulation. Thus, all simulations are run *n* times leading to randomly distributed results, as shown in section "Statistical considerations". If not stated otherwise, *n* = 300 is used for all investigated scenarios and simulation setups. All simulations are run using the same parameters with the only difference being the insertion seed used within LIGGGHTS® as initialization for the random number generator. Consequently, the only difference between the runs is where and in which orientation the parcels are inserted into the simulation domain. However, due to the random placement, the total number of parcels within the simulation domain also slightly varies depending on the used seed. All DEM simulations are analyzed in terms of different characteristics, all of which are described in the following sections "General definitions" and "Normalization".

The heap mass fraction ${\upomega }_{heap}$ is defined as the relative mass fraction of particles present in the heap to the total mass of particles after completing the drawdown. As the particles used in this work are monodisperse, this can also be formulated using the number of particles ${N}_{particles,i}$ in this respective domain *i* (*i* = tot: total number of particles in simulation domain, *i* = heap*:* number of particles in the heap after drawdown) as stated in Eq. (2-14).

2-14 $${\upomega }_{heap}=\frac{{N}_{particles,heap}}{{N}_{particles,tot}}$$

Generally, the simulation runs need to be classified into (i) cases where arching occurs and (ii) cases where arching does not occur. To achieve this in an automated fashion, the number of particles in a specific region within the simulation domain is counted and compared to an adjustable threshold, above which the case is counted as “arched”.

Specifically, this region is defined using the coordinate boundaries -0.03 $<x<$ 0.03 and z > 0.45 (and the full range of y positions). Doing so, only the center region (in the x-direction) of the upper box is considered as the relevant region for detection of arching. The detection region is indicated in Fig. 1a. The threshold is, following preliminary tests, set to 10. This ensures that only cases where arching really occurs are counted and artifacts within single simulation runs do not falsely exceed this threshold.

The relative number of arched cases is another important metric used in this work. After successful binary classification using the method outlined above, the so-called arching fraction ${\upeta }_{arch}$ is defined as stated in Eq. (2-15) using the number of arched simulation cases ${n}_{arch}$ and the total number of simulations $n$ for a specific set of parameters.

2-15 $${\upeta }_{arch}=\frac{{n}_{arch}}{n}$$

### Normalization

To be able to compare the results of our present work to other research and apply it to different geometries, the data needs to be non-dimensionalized. In addition, we normalize some important dimensionless quantities with reference quantities. This further helps to improve the physical interpretation of our results.

Using the normalization as stated in Eq. (2-16), one is able to compare different distributions more easily in terms of ${\upomega }_{heap}$.

2-16 $${\upomega }_{heap,norm}=\frac{{\upomega }_{heap}}{\overline{{\upomega }_{heap}}}$$

The gap width, critically affecting heap formation, is non-dimensionalized using a typical tetrapod size as given by Eq. (2-17).

2-17 $${N}_{parcels}=\frac{{W}^{*}}{\sqrt{8}\cdot {\xi }^{*}+{D}^{*}}$$

The denominator represents the edge length of a tetrapod with spheres on all vertices. The resulting number ${N}_{parcels}$ states how many parcels fit into a given gap with width ${W}^{*}$, thus providing a non-dimensional formulation for the tetrapod size. For all simulations done in this work ${W}^{*}$ = 0.25 m applies, as defined in section "Test setup".

Similarly, the number of spheres per gap width ${N}_{S}$ can be used to non-dimensionalize the sphere size ${D}^{*}$ used in our simulations. The resulting relation is shown in Eq. (2-18). It quantifies how many spheres of diameter ${D}^{*}$ fit into the gap width ${W}^{*}$. As ${W}^{*}$ is constant in our simulations, this formulation can be used directly to compare different ${D}^{*}$.

2-18 $${N}_{S}=\frac{{W}^{*}}{{D}^{*}}$$

Another parameter frequently used to assess DEM simulations or granular structures in general, is the particle volume fraction ${\phi }_{p}$, as defined in Eq. (2-19). The parcel volume ${{V}_{parcel}}^{*}$ is defined as the total volume of the four spheres that comprise the parcel. The volume of the box ${{V}_{box}}^{*}$ defines the total volume of the upper box, with the corresponding dimensions defined in section "Test setup". In case this box is filled with tetrapods, we calculate the particle volume fraction via:

2-19 $${\phi }_{p}=\frac{{N}_{parcels}\cdot {{V}_{parcel}}^{*}}{{{V}_{box}}^{*}}$$

Note that the above definition of ${\phi }_{p}$ is also meaningful in case tetrapods compenetrate each other (since the rods are virtual). This is since the tetrapods’ spheres overlap only minimally as governed by the force model defined in section "Force model" The void fraction $\varepsilon$, as defined in Eq. (2-20), and used later for comparison with Rolland et al.^15^, has to be normalized as the range of the expected void fraction results is very different. For normalization, Eq. (2-21) is used.

2-20 $$\varepsilon =1-{\phi }_{p}$$

2-21 $${\varepsilon }_{norm}=\frac{\varepsilon }{{\varepsilon }_{max}}$$

The insertion box width ${B}^{*}$ is normalized using its reference value ${B}_{0}^{*}$= 0.495 m (i.e., the physical upper box width in x-direction), as stated in Section "Test setup", using Eq. (2-22).

2-22 $${B}_{norm}=\frac{{B}_{0}^{*}}{{B}^{*}}$$

The effects of the latter dimensional characteristic of the simulation domain on the results are shown in the Supplementary Information (see Fig. A-3).

## Results

**Fig. 2:**
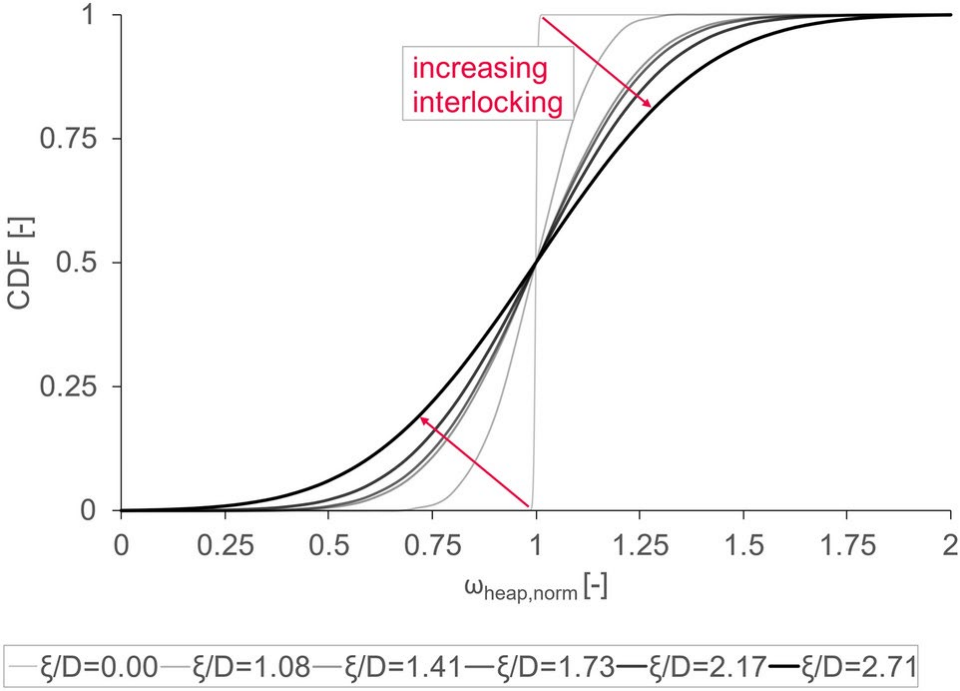
Cumulative density function CDF of the fitted distribution for six different *ξ/D*-ratios representing six different tetrapod shapes at constant ${N}_{S}=31.3$ using ${n}_{arch}$ simulation of arched cases (i.e., ${n}_{arch}=300\cdot {\upeta }_{arch}$) over normalized heap mass fraction ${\omega }_{heap,norm}$; the red arrows represent the direction of increasing interlocking strength.

A very important factor investigated in this work is the wide confidence interval of the found experimental distributions. The corresponding experimental results for a range of gap widths ${W}^{*}$ are provided in the Supplementary Information (see Figure A-1), which illustrates the inherent variability of the system used. As already pointed out in section "Statistical considerations", also our DEM simulations confirm significant variability, and suggest that fits based on normal distributions with mean µ and standard deviation $\sigma$ adequately describe the data. To deal with such distributions, it is necessary to find parcels showing a similar distribution and behavior in our simulations. As previously outlined, we use the tetrapod concept of Radl et al.^8^, as a basis for our DEM simulations. As the main feature of such an interlocking tetrapod parcel is its shape, it seems straight forward to investigate the impact of said characteristic first. To illustrate the effect of different parcel shapes exemplarily, the cumulative density function (CDF) of ${n}_{arch}$ simulations, where ${n}_{arch}$ is the number of arched cases among 300 simulations (i.e., ${n}_{arch}=300\cdot {\upeta }_{arch}$), for six different tetrapods shapes is shown in Fig. 2. The 300 simulations only differ in terms of the insertion seed used. One can clearly see that the slope of the fitted CDF gets smaller with increasing $\upxi /D$-ratio (i.e., stronger degree of interlocking, see our discussion in section "Parcel Size Effect"). Thus, the width of the respective distribution increases. This indicates a direct interplay between the variability of the DEM simulation and the interlocking potential of the used parcels. In the following sections, this hypothesis is thoroughly investigated.

## Effect of insertion parameters

### Location and rotational orientation

**Fig. 3:**
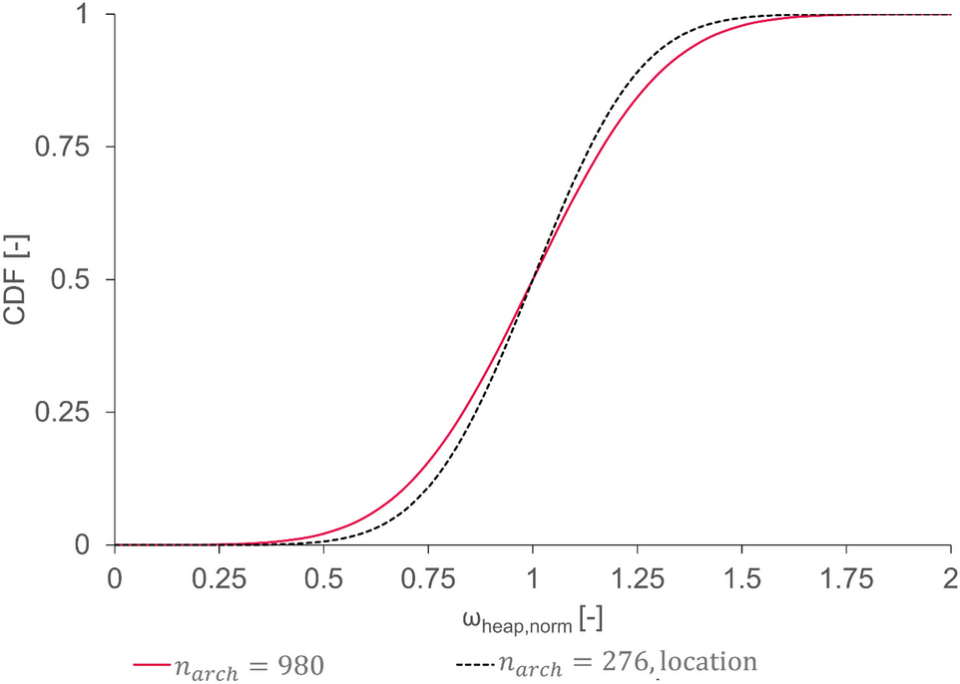
Cumulative density functions for tetrapods with *Nₛ* = 31.3 and $\upxi /D$ = 2.17 using ${n}_{arch}$ repetitions; varying location and rotational orientation (standard implementation, red continuous line) and only location (dashed black line) versus the normalized heap mass fraction ${\omega }_{heap,norm}$.

If not stated otherwise, the insertion seed is, as outlined in section "General definitions", the only input factor differentiating $n$ simulation runs from each other in the following sections. This being said, the insertion seed is used within LIGGGHTS® to initialize the random number generator for both the location and the rotational orientation of the inserted parcel. Thus, the individual effect of these two factors needs to be investigated first. To do so, $n$ simulations were performed with (i) both location and orientation and (ii) only the location being based on the random insertion seed. The fitted CDF for arched cases ${n}_{arch}=n\cdot {\upeta }_{arch}$ of these simulations is shown in Fig. 3. When comparing the two fits, one can identify that the slope of the CDF is mainly due to the random location. This suggests that the variability within the simulation results is based on the impact of the location on the interlocking behavior of the parcels. Nevertheless, to cover the full variability impact, the remainder of the DEM simulations is conducted considering both random location and orientation initialization.

### Parcel size effect

When looking at the variability of a DEM simulation, the size of the used parcel or particles is of high relevance. When using tetrapods, a parcel can be conclusively defined by stating the tetrapod radius ${\xi }^{*}$ and the diameter of the spheres in the tetrapod ${D}^{*}$. Consequently, the ratio of these two values, *ξ/D,* defines the shape of a given tetrapod, and hence its interlocking tendency. In principle, the geometry defines the interlocking tendency of the tetrapod irrespective of the scaling (i.e., size) of a tetrapod. However, the DEM simulation in this work used a fixed gap width ${W}^{*}$ for the drawdown tester, thus changing the relative size of the tetrapod in regard to the simulation domain when changing ${\xi }^{*}$ or ${D}^{*}$.

**Fig. 4:**
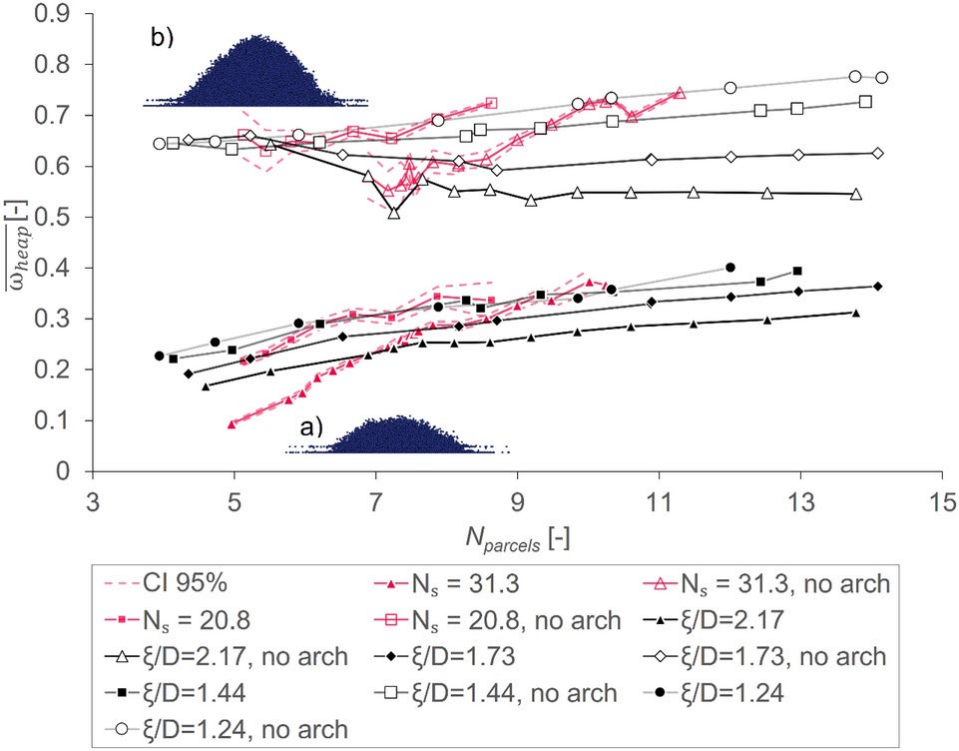
Average heap mass fraction $\overline{{\omega }_{heap}}$ versus number of parcels ${N}_{parcels}$ for arched cases (filled symbols) and non-arched cases (open symbols) for constant ${D}^{*}$ (i.e., ${N}_{S}$ = 31.3 and ${N}_{S}$ = 20.8) and constant ξ/D-ratios; a non-zero confidence interval exists only for constant ${D}^{*}$ cases and is indicated with dashed lines represent the confidence interval (CI, at a confidence level of 95%), only mean values $\overline{{\upomega }_{heap}}$ for all other cases are reported. The insets show snapshots of typical simulation result for (**a**) $\overline{{\omega }_{heap}}\approx 0.2$ and (**b**) $\overline{{\omega }_{heap}}\approx 0.6$.

As outlined in section "General definitions", the main characteristic feature that we look at in this work is the average heap mass fraction $\overline{{\upomega }_{heap}}$. To investigate the influence of the main elements defining the geometry of a given tetrapod, four different *ξ/D*-ratios and two different values for ${N}_{S}$ were investigated using a large range of ${N}_{parcels}$. To describe the simulation results adequately, the data was split up into arched and non-arched cases, as outlined in section "General definitions". The summary of the gained results, containing the information of about 22,000 DEM simulations in total, is shown in Fig. 4.

For arched cases, the effect of the *ξ/D*-ratio is consistent across the whole range of investigated number of parcels per gap width ${N}_{parcels}$. This indicates, that the bulkier the tetrapods get (i.e., the smaller the *ξ/D*-ratio gets and thus the spheres get bigger in relation to the rod length of the tetrapod), the larger the average heap mass fraction $\overline{{\upomega }_{heap}}$ becomes, irrespective of the parcel size. Furthermore, implying that smaller *ξ/D* ratios imply a smaller interlocking tendency. This leads to a larger fraction of particles falling down into the lower box. Thus, one can postulate a Hagen-Beverloo-like behavior in case of arching (i.e., the correlation between *N*_*parcels*_ and ${\omega }_{heap}$ is positive)^40^. A smaller tetrapod (i.e., a larger value of ${N}_{parcels}$) leads to a larger value for $\overline{{\upomega }_{heap}}$, indicating that interlocking alone cannot fully prevent parcels from falling down: there is a monotonic increase of $\overline{{\upomega }_{heap}}$ with ${N}_{parcels}$. Unfortunately, A saturation value for ${N}_{parcels}$ could not be identified from our simulations which would further support the Hagen-Beverloo-like behavior we observe.

For constant ${D}^{*}$ (i.e., ${N}_{S}$ = 31.3, ${N}_{S}$ = 20.8), the influence of the tetrapod radius ${\upxi }^{*}$ seems to be pronounced, especially at larger overall tetrapod sizes (i.e., ${N}_{S}$ = 20.8). This indicates that the interlocking tendency is strongly influenced by this characteristic. Additionally, smaller spheres lead to stronger interlocking compared to larger ones (compare: ${N}_{S}$ = 31.3 to ${N}_{S}$ = 20.8) at identical ${N}_{parcels}$. The slope of these two curves is significantly steeper than for the cases with constant *ξ/D*-ratios. This indicates a strong impact of the absolute values of ${\upxi }^{*}$ and ${D}^{*}$ on the outcome of a drawdown test simulation.

For non-arched cases, the values for $\overline{{\upomega }_{heap}}$ generally stay above 0.45 (see open symbols in Fig. 4). However, there seems to exist a trend towards more particles falling down into the heap for less interlocking parcels (i.e., $\upxi /D$= 1.24 or 1.44) and less falling down for stronger interlocking ones (i.e., $\upxi /D$ = 1.73 or 2.17). Thus, our simulations in case no arching occurs only partly follow a Hagen-Beverloo-like behavior (i.e., the correlation between *N*_*parcels*_ and $\overline{{\omega }_{heap}}$ is not strictly positive)^40^. The root cause of this interesting behavior could not fully be identified in our current work, and was postponed to a future analysis.

Furthermore, similar to the situation for arched cases, for constant ${D}^{*}$ (i.e., ${N}_{S}$ = 31.3, ${N}_{S}$ = 20.8), the tetrapod radius ${\xi }^{*}$ strongly influences $\overline{{\omega }_{heap}}$. This leads to a larger fraction of parcels falling down into the heap for smaller tetrapods. This further supports the hypothesis that interlocking is heavily influenced by ${\xi }^{*}$.

Another important parameter within every DEM simulation is the particle volume fraction ${\phi }_{p}$ (and the void fraction $\varepsilon$) as defined in Eq. (2-21). In our simulations, this was measured prior to the “drawdown test” (i.e., before opening the flap) from the number of parcels in the top region of the tester. We note in passing that in our present work the particle density ${{\uprho }_{p}}^{*}$ was not a variable. Thus, with changing ${\phi }_{p}$, the bulk density ${{\uprho }_{b}}^{*}$ would linearly change. In future calibration efforts, it is certainly possible to adjust the particle density to fit a measured bulk density. However, this is a comparably trivial extension, and hence this though was not further followed by us.

The particle volume fraction ${\phi }_{p}$ is documented for different $\upxi /D$-ratios and constant ${N}_{S}$ in the Supplementary Information, see Figure A-4. This data shows a clear trend towards higher values for smaller and hence bulkier parcels (i.e., smaller $\upxi /D$ values). However, the highest value observed for ${\phi }_{p}$ was 0.25 for $\upxi /D$ = 1.24, which can still be considered rather loose. This is due to the nature of the considered parcels, being connected by “virtual” rods with open space in between.

**Fig. 5:**
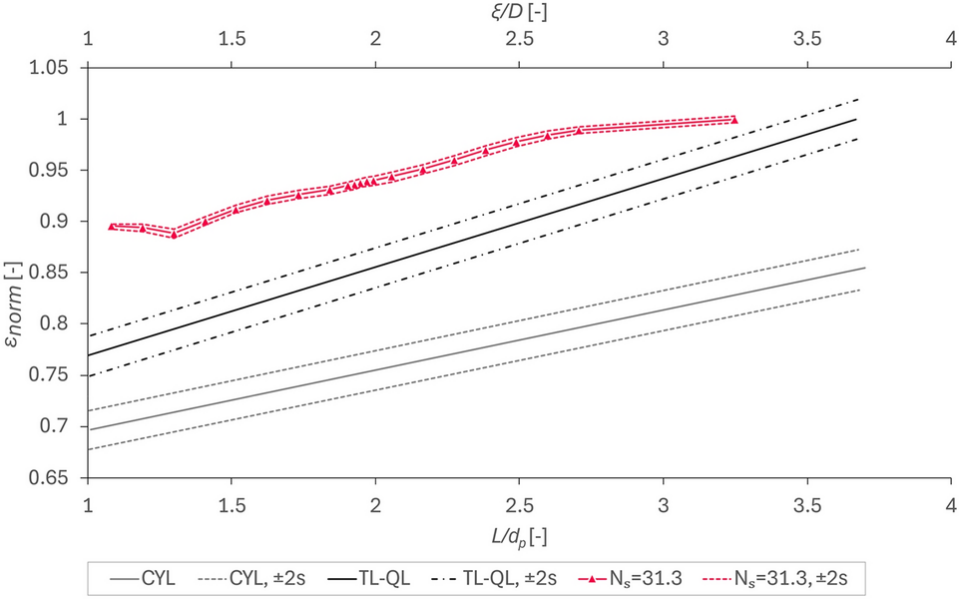
Normalized void fraction ${\varepsilon }_{norm}$ versus L/d_p_ for Rolland et al.^15^ (Fig. 7) on the primary axis and ξ/D for our present work using data from Figure A-4 and Eq. (2-22) to convert and normalize the data on the secondary axis.

The void fraction $\varepsilon$ is normalized using Eq. (2-22) and used to compare the data gained in this work to that of Rolland et al.^15^. In their work, they compare trilobes (TL), quadrilobes (QL), and cylinders (CYL) in terms of $\varepsilon$ at different ratios of $L/{d}_{p}$. The former two particles are, just like the tetrapods used in this work, non-convex. In their work, the ratio is slightly differently defined compared to our present work. However, due to the shape of the used tetrapods and the missing “diameter” of the whole parcel, using $\upxi /D$ as a comparable ratio was deemed sufficient. Thus, the data published by Rolland et al.^15^ (black), more specifically of their Fig. 7, is shown in conjunction with our present results (red) in Fig. 5.

Especially when compared to the TL-QL-approach, the tetrapods yield a similar slope at a much narrower “confidence interval”, as defined by Rolland et al.^15^ to be equivalent to $\overline{x }\pm 2s$. This discrepancy is likely to be associated to the much higher number of samples used in our present work: specifically, we used *n* = 300 per datapoint (i.e., 5700 simulations for the 19 datapoints), whereas Rolland et al.^15^ used just 19 datapoints in total without repetitions of individual simulations. Other than that, our data agrees, after normalization, with the trend observed by Rolland et al.^15^, just at a higher level of the void fraction.

### Arching behavior

**Fig. 6:**
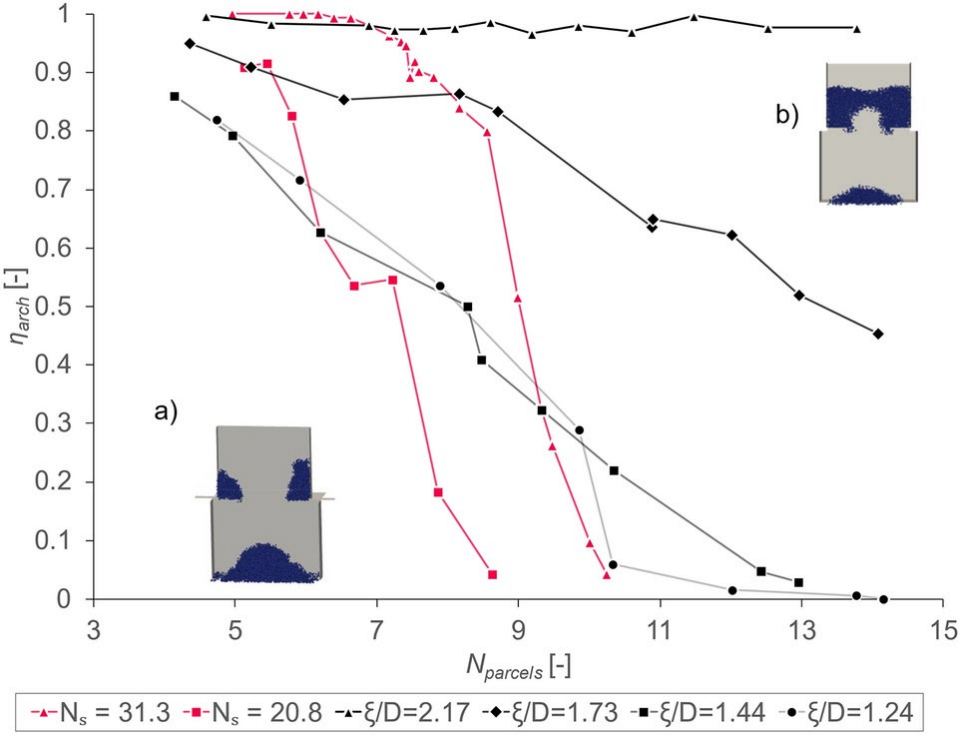
Arch fraction ${\eta }_{arch}$ over number of parcels ${N}_{parcels}$ for constant ${D}^{*}$ (*N*_*S*_ = 31.3, *N*_*S*_ = 20.8) and constant $\xi /D$ fractions; insets show typical simulation results for (**a**) no arching and (**b**) arching.

As previously outlined, the analysis of the average heap mass fraction $\overline{{\upomega }_{heap}}$ as main characteristic property of the drawdown test requires splitting up the simulations into “arched” and “non-arched” cases. This approach was already described in section "General definitions". However, it might also be useful to look at the relative probability of occurrence within this binary classification method. Thus, the arching fraction ${\eta }_{arch}$, as defined in Eq. (2-15), is plotted versus a large range of ${N}_{parcels}$ for four different *ξ/D*-ratios and two different values for ${N}_{S}$ as shown in Fig. 6**.**

Quiet clearly, the arching fraction is strongly dependent on ${N}_{parcels}$. As this metric describes the relative tetrapod size compared to the gap width ${W}^{*}$, this relationship was to be expected. No matter how strong the interlocking, as defined by the $\xi /D$-ratio is, the smaller the parcels get, the more often no arch is formed due to the gap getting relatively speaking “bigger”.

However, the $\xi /D$-ratio has a strong influence on the slope of this descending trend. This influence is especially pronounced for $\xi /D$ = 2.17, as ${\upeta }_{arch}$ seems to be independent of ${N}_{parcels}$ in the investigated range. Though, this might only be due to the fact, that said range of ${N}_{parcels}$ is too small to observe an effect (note that large values for ${N}_{parcels}$ require more extreme computational resources, which are intractable considering the large number of repetitions of simulations). For smaller values of $\xi /D$, the increasingly steep slope is clearly visible. This suggests that the level of interlocking could be used as a proxy to set a desired arching fraction ${\upeta }_{arch}$, based on experimental results, to model a real system adequately. At constant sphere radii ${D}^{*}$ (i.e., ${N}_{S}$ = 31.3, ${N}_{S}$ = 20.8), the slope is noticeably steeper, suggesting—same as in the case of ${\upomega }_{heap}$ previously shown in section "Parcel Size Effect"—that the influence of the tetrapod radius ${\upxi }^{*}$ seems to be pronounced. Knowing this, one can use both $\xi /D$ exclusively as well as in conjunction with ${D}^{*}$ and ${\xi }^{*}$ to calibrate ${\upeta }_{arch}$ to experimental values, depending on the needs of each individual experiment.

### Effect on variability

To be able to accurately implement the method outlined in the previous sections, it is necessary to be able to measure the amount of spread within $n$ simulation runs. Thus, the width of the underlying distribution can be quantified to be used as a valuable prediction of the simulation. As pointed out in section "Statistical considerations", using normal distributions for this purpose is a sound assumption given our preliminary testing results. To assess the width of said distribution, the index of dispersion *ID*, as defined in Eq. (2-12), is a useful metric. The value which is used for generating the distributions is ${\omega }_{heap}$ for each of the simulations.

**Fig. 7:**
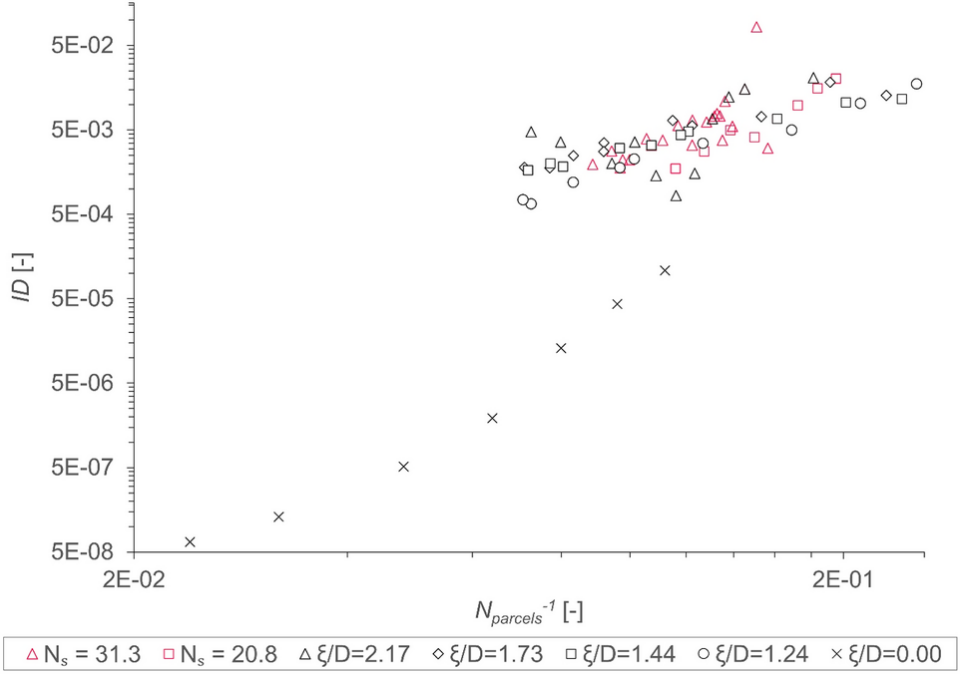
Index of dispersion *ID* over reciprocal number of parcels in the simulation domain ${{N}_{parcels}}^{-1}$ with constant ${N}_{S}$ and with constant *ξ/D*-ratios using data of non-arched cases.

*ID* is plotted over a large range of ${{N}_{parcels}}^{-1}$ for five different $\xi /D$-ratios and two different values for ${N}_{S}$ as shown in Fig. 7. However, for $\xi /D$ = 0 this becomes misleading, as the parcel just consists of one sphere in this case. Thus, the data for $\xi /D$ = 0 considers simulations with considerably fewer particles at the same value of ${{N}_{parcels}}^{-1}$, compared to the other simulations. Consequently, this implies a very different computational expense, at the same value of the abscissa. This has to be considered when evaluating the results gained here. In addition, only non-arched cases were used for this analysis, as $\xi /D$ = 0 (i.e., spheres) only yields non-arched cases.

Nevertheless, *ID* decreases for all cases with increasing ${N}_{parcels}$, which indicates, that a bigger sample size (i.e., larger number of ${N}_{parcels}$) reduces the overall variability. This finding nicely agrees with the well-established law of large numbers found in all branches of science. In addition, *ID* increases with $\xi /D$ to some degree, indicating a proportional effect of interlocking on the variability of our simulation results. As interlocking—due to its high dependence on the initial positions of tetrapods—can be described as the main factor for chaotic behavior in the simulations used in this work, this finding intuitively makes sense.

The tetrapod radius ${\xi }^{*}$, as was previously found in sections "Parcel Size Effect" and "Arching Behavior" for $\overline{{\omega }_{heap}}$ and ${\upeta }_{arch}$, respectively, seems to be of high importance for each metric of the simulation, including the *ID*. This may be due to its impact on the interlocking tendency of the parcels.

**Fig. 8:**
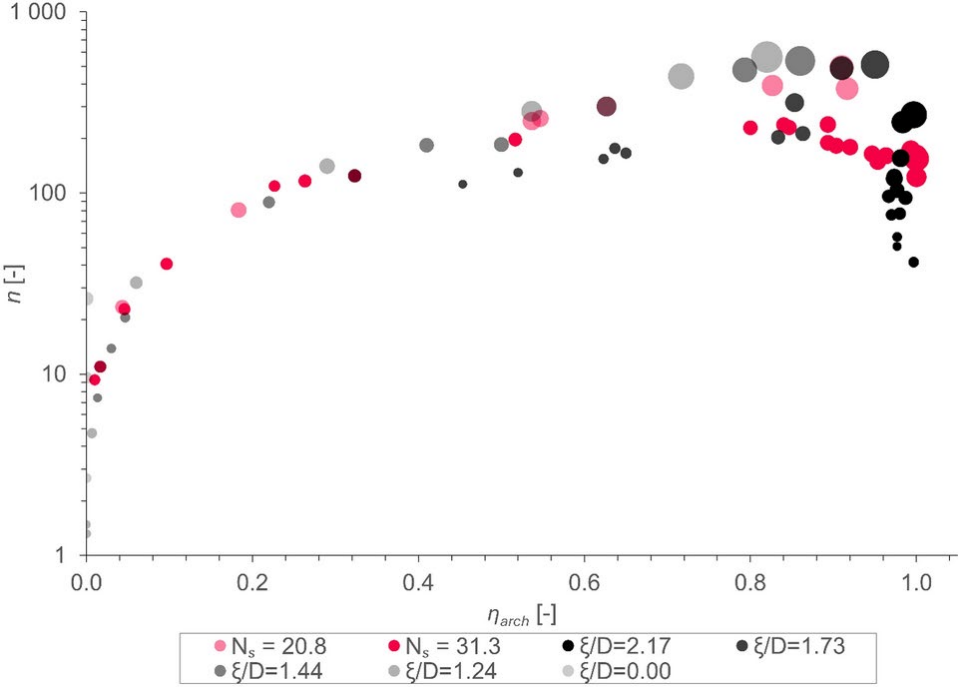
Number of required simulations $n$ over ${\upeta }_{arch}$ for tetrapods with constant ${N}_{S}$ and constant $\xi /D$-ratio; the size of the marker represents ${{N}_{parcels}}^{-1}$: the larger the tetrapod, the bigger the marker.

Using the previously explained assumption of a normal distribution of the gathered data, the required number of samples to reach a certain degree of confidence can be established. In this regard, a 95% confidence level and a width of $\overline{x }\pm \left(\overline{x }\cdot 5 \%\right)$ is set as the threshold. The mathematical relationship used in this case is shown in Eq. (2-13). The required number of samples $n$ versus ${\upeta }_{arch}$ is shown in Fig. 8.

In this case, the whole population is used without previous classification into arched and non-arched cases. However, note that arching is implicitly considered due to $\overline{x }$ and $s$ being influenced by it (i.e., for moderate ${\upeta }_{arch}$, $s$ can never get small, for values at or near 0 or 1 it naturally is smaller). To investigate edge cases (e.g., for ${\upeta }_{arch}\approx 0.999$ the non-arched case) with a narrow confidence interval, a much higher number of samples would be needed due to the low probability of occurrence of such a case. Trivially, this number could be estimated by the inverse of the probability of occurrence of such a case. Further investigation into this issue is not the focus of our present work, and is thus not considered.

Using this formulation, one can identify a clear trend towards higher $n$ for larger tetrapods, indicated by bigger markers, and higher ${\upeta }_{arch}$. Reiterating the concept of the law of large numbers, the former intuitively makes sense: the more parcels are located within the simulation domain, the smaller the impact of a single, chaotic, event actually is. As already pointed out prior to this, one of the hypotheses considered in this work is concerned with the effect of interlocking on the variability of a DEM simulation. As the amount of interlocking is clearly positively correlated with ${\eta }_{arch}$, the data shown in Fig. 8 underpins our already stated hypotheses: the stronger the interlocking and the bigger the parcels, the higher the variability and thus the number of samples needed to achieve the targeted (i.e., narrow) confidence interval.

### Application

To illustrate a potential strategy to apply the outlined concept, three testcases with specifications shown in Table 2 are considered.

**Table 2:** Specification of testcase in terms of target values for $\overline{{\upomega }_{heap,noarch}}$ and *ID*.

Case	Parameter [-]	Value
1	$\overline{{\upomega }_{heap,noarch}}$	0.70
	$ID$	${1}\cdot {10}^{-3}$
2	$\overline{{\upomega }_{heap,noarch}}$	0.65
	$ID$	${2}\cdot {10}^{-3}$
3	$\overline{{\upomega }_{heap,noarch}}$	0.57
	$ID$	${5}\cdot {10}^{-3}$

By using the data presented in sections "Arching Behavior" and "Effect on variability" and taking either the raw data, or fitting the data across all investigated ratios of $\upxi /D$, one can arrive at three-dimensional surface plots for either (i) $\upxi /D$ (x), ${N}_{parcels}$ (y), $\overline{{\upomega }_{heap,noarch}}$ (z), or (ii) $\upxi /D$ (x), ${N}_{parcels}$ (y), $ID$ (z). For the simulation data shown in Figs. 6 and 7, the best fits are achieved when using a regression with polynomial features up to degree two. By normalizing the z-axis of both plots (i.e $., \overline{{\upomega }_{heap,noarch}}$ or $ID$) by the respective target values stated in Table 2, the intersection of both normalized surface plots at $z=1$ yields the required values for $\upxi /D$ and ${N}_{parcels}$ for a given parameter set. Exemplarily the resulting intersected plot for case 2 can be found in the Supplementary Information (see Figure A-5).

The results can then be validated by running DEM simulations with the respective parameter values for ${N}_{parcels}$ and $\upxi /D$. The results of said simulations for the original dataset, in conjunction with the parameter values used, are shown in Table 3.

**Table 3:** $\upxi /D$ and ${N}_{parcels}$ for testcases with the parameters as stated in Table 2, validation parameters ($\overline{{\upomega }_{heap,noarch}}$ and $ID$) based on simulation data using the stated values for $\upxi /D$ and ${N}_{parcels}$, and the respective relative error to target values; Comparison of (i) original dataset with polynomial degree 2, (ii) original dataset with optimal polynomial degree using cross-validation, and (iii) using an extended dataset.

		Original Dataset		Cross-Validated Optimal Polynomial Degree		Extended Dataset	
Case	Parameter [-]	Value	Relative Error [%]	Value	Relative Error [%]	Value	Relative Error [%]
1	$\upxi /D$	1.26		1.26		1.26	
	${N}_{parcels}$	10.24		10.00		10.00	
	$\overline{{\upomega }_{heap,noarch}}$	0.73	+ 4	0.72	+ 3	0.72	+ 3
	$ID$	$\text{2.2}\cdot {10}^{-3}$	+ 120	$\text{2.3}\cdot {10}^{-3}$	+ 129	$\text{2.3}\cdot {10}^{-3}$	+ 129
2	$\upxi /D$	1.44		1.42		1.79	
	${N}_{parcels}$	8.48		8.72		10.93	
	$\overline{{\upomega }_{heap,noarch}}$	0.67	+ 3	0.68	+ 4	0.61	-6
	$ID$	$\text{4.4}\cdot {10}^{-3}$	+ 118	$\text{3.1}\cdot {10}^{-3}$	+ 54	$\text{3.0}\cdot {10}^{-3}$	+ 52
3	$\upxi /D$	2.02		2.02		2.02	
	${N}_{parcels}$	9.57		9.50		9.50	
	$\overline{{\upomega }_{heap,noarch}}$	0.57	0	0.57	+ 1	0.57	+ 1
	$ID$	$\text{5.4}\cdot {10}^{-3}$	+ 8	$\text{4.8}\cdot {10}^{-3}$	− 4	$\text{4.8}\cdot {10}^{-3}$	− 4

The original dataset comprises of the data shown in Figs. 4 and 7, while the extended dataset includes additional datapoints to improve the accuracy of the validation results in the vicinity of the testcases.

Considering the accuracy with respect to $\overline{{\upomega }_{heap,noarch}}$, the used regression and intersection yields reasonably accurate results with errors up to 4% in the considered cases. However, the sensitivity of the simulation regarding the width of the distribution, in this respect expressed as *ID*, seems to be considerable. Thus, using a simple polynomial fit with features up to degree two, the resulting error is considerably higher. Nevertheless, we are able to achieve values in the same order of magnitude for *ID*.

Considering the gained results, the data presented in this work can be used to find—given sufficiently interlocking particles—fitting parameters for tetrapods to represent the real system in DEM simulations. To get more accurate results, the regression may be refined or the resolution of datapoints may be upscaled. The former can be achieved by finding the respective optimal polynomial degree for both surfaces using *k*-fold-cross-validation^41^ (see the data in the middle column in Table 3). The latter approach is exemplarily illustrated by performing additional simulations using parameter values in the vicinity of the ones shown in Table 3 (rightmost column) for the original datasets. Using both methods, clearly, improves the accuracy in terms of *ID* for testcase 2 as is shown in Table 3. In testcase 1 we were not able to achieve an improvement: considering the low target *ID* (compared to what is shown in Fig. 7), testcase 1 seems to be located at the edge of the investigated subspace. Consequently, the low number of training data in this subspace poses a challenge to match the target *ID*. As the focus of our present work was to present a new concept of looking at calibration parameters for a DEM simulation, the generation of more training data was not followed. Such an effort would require a significantly large computational expense, but not introduce a fundamentally new concept.

To compare the data gained with the specifications in Table 2 to a (i) cohesionless (i.e., $${k}_{sjkr}^{*}=0)$$ and (ii) cohesion- and frictionless (i.e., $${k}_{sjkr}^{*}=0$$, $${\mu }_{rs}=0$$, $${\mu }_{rr}=0$$) setup, we performed additional validation runs. The results of these simulations are stated in Table 4. Evidently, the impact of friction on our results is significant. While the lack of cohesion as in (i) only increases $$\overline{{\upomega }_{heap,noarch}}$$ by $$\sim 10 \%$$, we observed a drastic increase in the cohesion- and frictionless case (ii). Specifically, case (ii) lead to a nearly complete drawdown of all particles within the simulation domain. Thus, friction is important for the physical interlocking of tetrapods. Oppositely, using a cohesionless system should, based on these three testcases, be possible, again not introducing a fundamentally new concept. Thus, we omitted exploring this option further in our present work.

**Table 4:** $\upxi /D$ and ${N}_{parcels}$ for testcases with the parameters as stated in Table 2, validation parameters ($\overline{{\upomega }_{heap,noarch}}$ and $ID$) based on the original dataset using the stated values for $\upxi /D$ and ${N}_{parcels}$ from Table 3 (i) without cohesion and (ii) neither with cohesion nor friction.

Case	Parameter [-]	No Cohesion	No Cohesion & Friction
1	$\overline{{\upomega }_{heap,noarch}}$	0.74	0.99
	$ID$	0.001	${2}\cdot {10}^{-6}$
2	$\overline{{\upomega }_{heap,noarch}}$	0.70	0.99
	$ID$	0.003	0.002
3	$\overline{{\upomega }_{heap,noarch}}$	0.64	0.99
	$ID$	0.004	${8}\cdot {10}^{-6}$

Finally, we have investigated the effect of other influencing factors on the outcome of our simulations, e.g., the particle–particle friction coefficient. However, all of these parameters were found to be of relatively minor importance, and hence are presented in the Supplementary Information (see Fig. A-6). The variables used in this analysis are stated in Table A-1.

## Conclusion

We simulated the flow of spherical and tetrapod-shaped parcels in a drawdown tester, which is often used to calibrate parameters of DEM simulators to real-world experimental data. Thereby, we were able to successfully show that strongly non-spherical and interlocking particles, such as tetrapods, introduce a significantly larger uncertainty into the simulation outcome compared to results when considering spherical particles. This uncertainty is pronounced in both of the main observed parameters: the heap mass fraction ${\upomega }_{heap}$ and the arching fraction ${\upeta }_{arch}$. Furthermore, the applicability of tetrapods to model interlocking behavior was conclusively demonstrated.

Firstly, we found that interlocking increases the width of the resulting distribution of the observed simulation (i.e., the uncertainty). Furthermore, the random location of the individual particles, in contrast to their orientation, was found to impact the resulting distribution more significantly.

Secondly, the influence of sphere diameter ${D}^{*}$ (for a fixed opening width) and the tetrapod shape (i.e., the $\xi /D$ ratio) was found to have a considerable impact on the average heap mass fraction $\overline{{\omega }_{heap}}$. Generally, larger $\xi /D$ ratios resulted in a smaller $\overline{{\omega }_{heap}}$, leading to more particles staying in the upper box. Due to stronger interlocking with increasing *ξ/D* ratio, this result intuitively makes sense. Tetrapods with a constant ${N}_{S}$ (i.e., constant sphere size) showed a stronger correlation between the relative size of the parcel ${N}_{parcels}$ and $\overline{{\omega }_{heap}}$. Smaller tetrapods show a Hagen-Beverloo-like behavior. For the non-arched case, the results were less conclusive, and Hagen-Beverloo-like behavior was not always observed.

Next, the arching fraction ${\eta }_{arch}$ was investigated across a range of ${N}_{parcels}$. Clearly, the smaller particles lead to less arching. However, the shape of the parcel, especially the *ξ/D*-ratio was found to be of key importance for the slope of this relationship: The higher *ξ/D,* the smaller the slope becomes, leading to no observable influence for the largest investigated *ξ/D* = 2.17 over the investigated range.

To really be able to judge the uncertainty, the index of dispersion *ID* was investigated for all simulations. It was shown that interlocking (i.e., bigger values of *ξ/D*) negatively influences *ID*, thus increasing uncertainty. Furthermore, a clear trend towards higher values of *ID* for smaller numbers of ${N}_{parcels}$ could be identified, which is supported by the law of large numbers. Thus, one would need small values for *ξ/D* and large values for ${N}_{parcels}$ (i.e., small parcels) to achieve small *ID*. This aligns with the concept of the tetrapod approaching spherical shape when reducing *ξ/D* until the spheres at the vertices show considerable overlap. However, in this study overlapping cases were not considered.

It was shown that larger parcels and stronger interlocking are the main contributing factors increasing the uncertainty. Thus, the number of samples $n$ needed to achieve a narrow confidence interval (CI) increases with increasing parcel size. Depending on the parcel size and ${\eta }_{arch}$, one needs to consider between 5 and 300 samples to get a narrow CI at reasonably small parcel size. Generally, less arching results in smaller *n*. Knowing this, it also becomes clear, why using small *n* (or single simulations) in other studies found in literature was not an issue: for non-interlocking spheres variability of the model output is simply very small.

Using this information, we proved that our findings can be used to give guidance on parcel size and shape for interlocking particles when provided with experimental data.

Summarizing, we managed to establish a conceptual framework for interpreting DEM simulation data of non-spherical particles statistically. Using this framework, we were able to discover the most relevant parameters for uncertainty and estimate their impact. By combining the absolute value of a goal parameter and the distribution width of an experimental result, we were able to suggest parcel properties for three given testcases based on the data gained during this study.

Using the data and framework presented in this work, the used concepts can be applied to other flow challenges, such as steady-state shear, or be extended to other parcel geometries. Another idea would be to deepen the analysis, e.g., to detect the arching-flow phase transition based on an alternative metric. Along this way of thoughts, it would be also interesting to extend our initial study of non-cohesive frictionless tetrapods: this could answer the question whether “purely” interlocking tetrapods also show this phase transition. Also, our present study focused on the phenomenological description of parcel properties influencing the uncertainty of DEM simulations. Potentially, however, one could also investigate the influencing factors on a bulk-level, e.g., using geometric distribution functions. One such approach was proposed by Holloway et al.^42^. In their contribution, they applied the radial distribution function (RDF) to analyze the structure of a sheared bed of particles. However, their approach could also be used to quantify the degree of interlocking in a particle bed such as the one when initializing the drawdown tester simulations as in our present study.

## Supplementary Information

Supplementary Information 1.

## Data Availability

All data used to generate the results within the manuscript is documented here: 10.3217/25g2e-6ha82.

## Abbreviations

COM: Center of mass
CI: Confidence interval
DEM: Discrete element method
HPC: High-performance computing
KS: Kolmogorov–Smirnov
N/A: Not available
sjkr: Simplified Johnson-Kendall-Roberts cohesion model

## Latin characters

${B}_{norm}$: Normalized size of box; [-]
${B}^{*}$: Size of box; [m]
$${c}^{*}$$: Damping coefficient; [$${\text{N}}\cdot {\text{s}}\text{/}{\text{m}}$$]
CDF: Number-based cumulative density function; [-]
${D}^{*}$: Diameter of spheres constituting a tetrapod; [m]
$$E$$: Error; [-]
${{f}_{i}}^{*}$: Force of type i; [N]
$${{\varvec{g}}}^{*}$$: Gravitational acceleration; [$$\text{m/}{\text{s}}^{2}$$]
${{\mathbf{I}}_{i}}^{*}$: Moment of inertia tensor of particle i; [${\text{kg}}\cdot {\text{m}}^{2}$]
$ID$: Index of dispersion; [-]
${{k}_{sjkr}}^{*}$: Cohesion energy density; [${\text{J}}\text{/}{\text{m}}^{3}$]
${L}^{*}$: Tetrapod edge length; [m]
${{m}_{i}}^{*}$: Mass of particle i; [kg]
$n$: Number of samples; [-]
${n}_{arch}$: Number of arched samples; [-]
${N}_{parcels}$: Number of parcels per gap width ${W}^{*}$; [-]
${{N}_{parcels}}^{-1}$: Reciprocal number of parcels in the simulation domain; [-]
${N}_{particles,i}$: Number of particles i in the simulation domain; [-]
${N}_{S}$: Number of spheres per gap width ${W}^{*}$; [-]
PDF: Number-based probability density function; [-]
${{r}_{i}}^{*}$: Radius of particle i; [m]
$s$: Sample standard deviation; [-]
${t}^{*}$: Time (or time step); [s]
${{\mathbf{t}}_{i}}^{*}$: Torque of type i, [N⋅m]
${t}_{1-\frac{\alpha }{2},\hspace{0.17em}n-1}$: Two-sided t-value based on student-t-distribution with confidence level $1-\alpha /2$ and $n-1$ degree of freedom; [-]
${W}^{*}$: Gap width of Drawdown Tester; [m]
${{v}_{i}}^{*}$: Velocity of particle i; [m/s]
$$\overline{x }$$: Sample mean; [-]
${{x}_{i}}^{*}$: Position of particle i; [m]
${Y}^{*}$: Youngs modulus; [Pa]

## Greek characters

$$\alpha$$: Significance level; [-]
$\upbeta$: KS-Test Goodness-of-Fit value; [-]
${\upbeta }_{\text{T}}$: KS-Test Goodness-of-Fit threshold value; [-]
$$\delta^\star$$: Overlap; [m]
$${{\delta }_{n}}^{*}$$: Normal overlap; [m]
$${{\delta }_{t}}^{*}$$: Tangential overlap; [m]
${\varepsilon }_{norm}$: Normalized void fraction (porosity); [-]
${\eta }_{arch}$: Fraction of simulation results with arching; [-]
${\upeta }_{arch,norm}$: Normalized fraction of simulation results with arching; [-]
$\mu$: True mean of normally distributed random variable; [-]
${\mu }_{rr}$: Rolling friction coefficient; [-]
${\mu }_{rs}$: Sliding friction coefficient; [-]
$${\xi }^{*}$$: Tetrapod radius; [m]
ξ/D: Interlocking parameter (i.e., tetrapod radius to sphere diameter ratio); [-]
$${{\uprho }_{b}}^{*}$$: Bulk density; [$$\text{kg}/{\text{m}}^{3}]$$
$${{\uprho }_{p}}^{*}$$: Particle density; [$$\text{kg}/{\text{m}}^{3}$$]
$\sigma$: True standard deviation of normally distributed random variable; [-]
${\phi }_{p}$: Particle volume fraction; [-]
${{\varphi }_{i}}^{*}$: Angular position of particle *i*; [rad]
$${\omega }_{i}^{*}$$: Angular velocity; [$$\text{rad/s}$$]
${\omega }_{heap}$: Particle heap mass fraction; [-]
$\overline{{\omega }_{heap}}$: Average particle heap mass fraction for *n* simulations; [-]
$\overline{{\omega }_{heap,noarch}}$: Average particle heap mass fraction in case on no arching for *n* simulations; [-]
${\omega }_{heap,norm}$: Normalized particle heap mass fraction; [-]

## Code availability

https://gitlab.tugraz.at/simSci/uncertaintyDEMSimulationsInterlocking.
