# Supplementary material for: The uncertainty inherent to DEM simulations of interlocking particles
Source: Sci Rep. 2025 Mar 4;15:7599. doi: 10.1038/s41598-025-90129-6 (PMC11880563; doi:10.1038/s41598-025-90129-6)
Supplement: Supplementary file 1 — Supplementary Information 1. [file 41598_2025_90129_MOESM1_ESM.pdf]

The Uncertainty Inherent to DEM Simulations of Interlocking Particles

Authors: Lukas Maier [1], Michael Mitterlindner [1], Hadie Benabchiasli [1], Gregor Fasching [1], Stefan Radl* [1]

Keywords: Discrete Element Method, Simulation, Non-Spherical Particles, Machine Learning

Affiliation: [1] Institute of Process and Particle Engineering, Graz University of Technology, Inffeldgasse 13/III, 8010 Graz, Austria

Contact Information: radl@tugraz.at

A Supplementary Information

A.1 Experimental Data

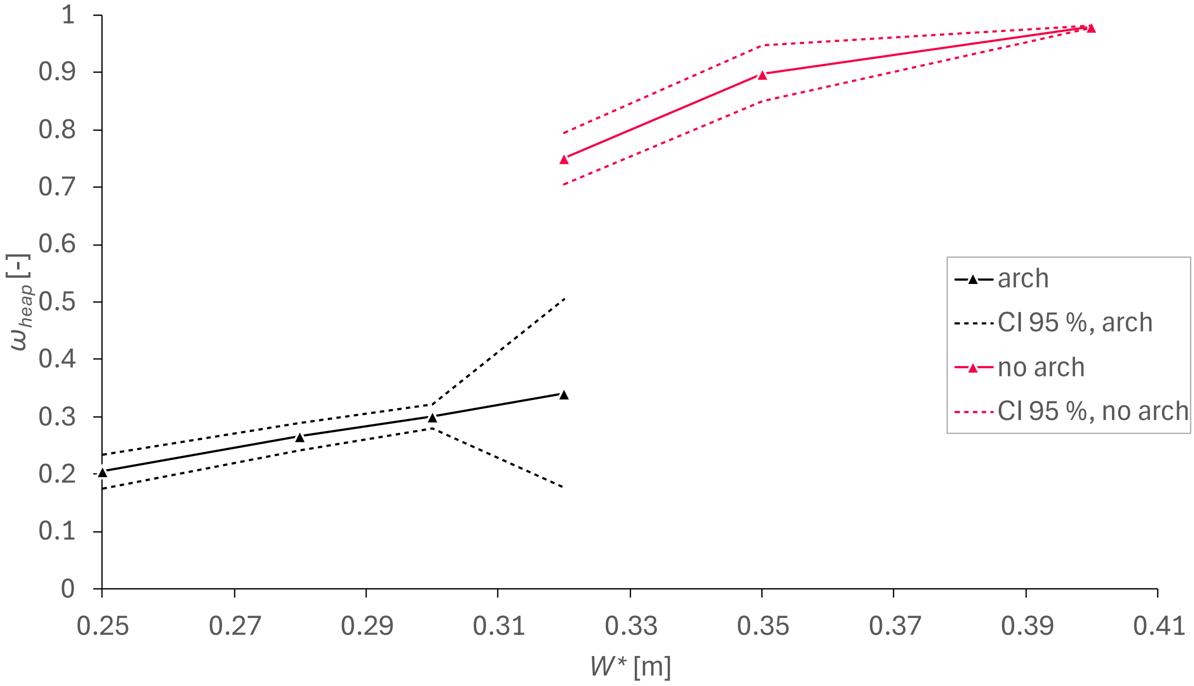

Figure A-1: Experimental data for the average heap mass fraction $\overline{\omega_{heap}}$ versus different gap widths W* with n = 6 for each of the investigated W*; data split up into arched data (black) and no arch data (red) with the respective confidence interval CI indicated using dashed lines.

A.2 Preliminary Testing

To be able to apply the methods outlined in section 2.4, one first needs to assess whether the underlying distribution is in fact a normal distribution. For this purpose, various standardized tests, such as the Kolmogorov-Smirnov (KS) – test, can be used [1]. Said test compares a fitted standard distribution with the number based cumulative density function (CDF) of the simulation data. The maximum absolute difference between the two distributions is evaluated. By comparing this value to a tabulated threshold-value dependent on the sample size *n*, one can test for validity of the

assumption of normal distribution of the data. Formally, this process can be described using Eq.(A-1), where *f(x)* is the CDF of the simulation data at position x and $n(x|\mu,\sigma^2)$ is the CDF of a normal distribution at position x with given μ and σ^2. The threshold value for a two-sided significance level α of 0.05 is given to be β_T = 0.043 for *n* = 980. For the investigated dataset using N_S = 31.3, ξ/D = 2.17, and *n* = 980 one arrives at β = 0.032 using this method. Thus, the null-hypothesis of the underlying distribution being a normal distribution is accepted.

$$\beta = \max(|f(x) - n(x|\mu,\sigma^2)|) \leq \beta_T \quad \text{(A-1)}$$

The simulation data as well as the corresponding normal distribution with fitted parameters is shown in Figure A-2.

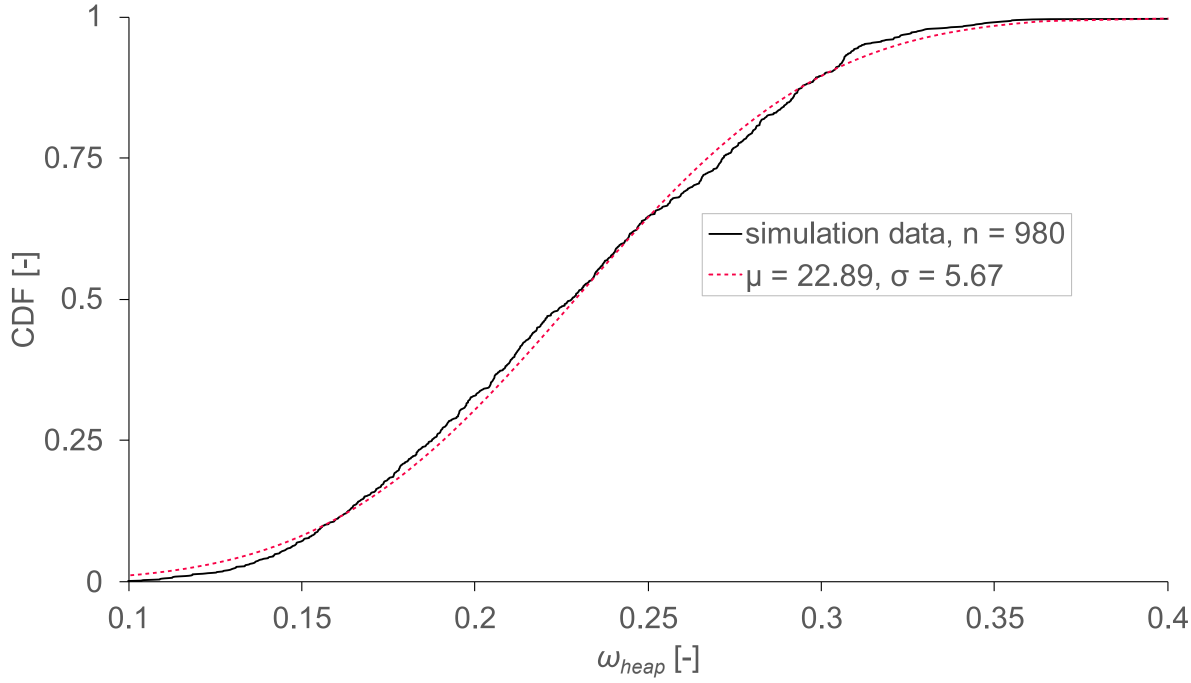

Figure A-2: Number based cumulative density function (CDF) versus heap mass fraction ω_{heap} for simulation data using N_S = 31.3, ξ/D = 2.17, and *n* = 980 (black) and fitted normal distribution with stated μ and σ (red dashed); Goodness-of-Fit test results: β = 0.032.

A.3 Insertion box size

The normalized size of the simulation box B_{norm} is another factor influencing the simulation results. The limiting case, $B_{norm} = 1$, was used in this present work, as this is how typical DEM simulations are setup. It represents a situation where parcels are inserted across the whole cross section of the upper box (i.e., $B = B_0^*$), resulting in wall effects potentially influencing the simulations by reducing ρ_b near the wall of a container. Thus, this factor was investigated as well. The results for *ID* using two different sizes of tetrapods (N_S = 31.3, N_S = 17.9) with different normalized box sizes B_{norm} are shown in Figure A-3.

Considering two different values of N_S (i.e., 17.9, and 31.3), the width of the resulting distributions decreased by 28 and 14 %, respectively, in case of a 5 % bigger physical box. This indicates, that, just as pointed out earlier, insertion near the wall can have a negative impact on variability. For N_S = 31.3, the effect seems limited to a narrow region

near the wall, as *ID* is constant for B_{norm}> 1.01. This indicates, that the size of the spheres D^* plays a crucial role for the “wall-effects”.

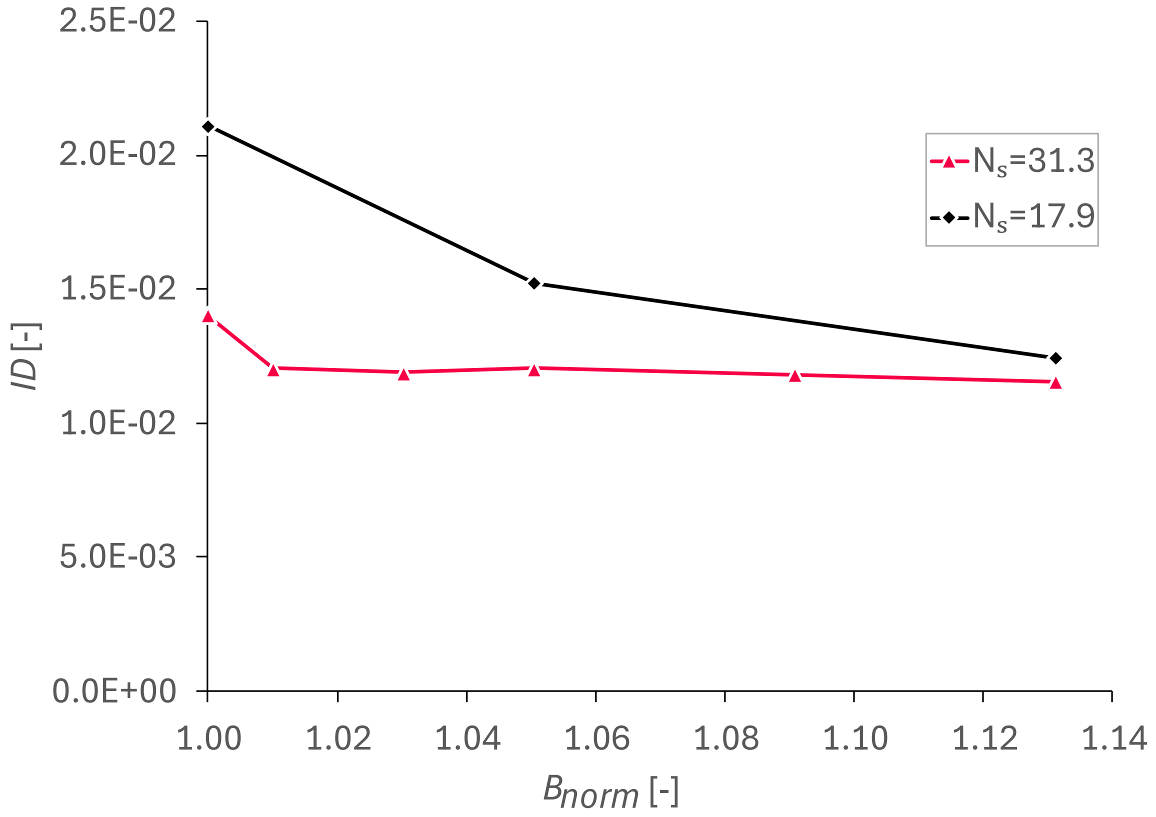

Figure A-3: Index of dispersion *ID* over normalized box size B_{norm} for smaller ($N_S = 31.3$) and bigger ($N_S = 17.9$) spheres used in tetrapod.

A.4 Effect on Porosity

To assess the impact of parcel size and shape on the porosity of the particle bed in the upper box after settling in the performed DEM simulation, the particle volume fraction ϕ_p, as defined in Eq. (2-19), is used. The mean observed particle volume fraction ϕ_p is documented for different ξ/D-ratios and constant N_S in Figure A-4. This data shows a clear trend towards higher values for smaller and bulkier parcels (i.e., smaller ξ/D values). However, the highest ϕ_p observed is at 0.25 for ξ/D = 1.24, which can still be considered rather loose. This is due to the nature of the considered parcels, consisting of spheres being connected by “virtual” rods with open space in between. The size effect is clearly caused by wall effects, similar to what we have seen in our insertion box size study.

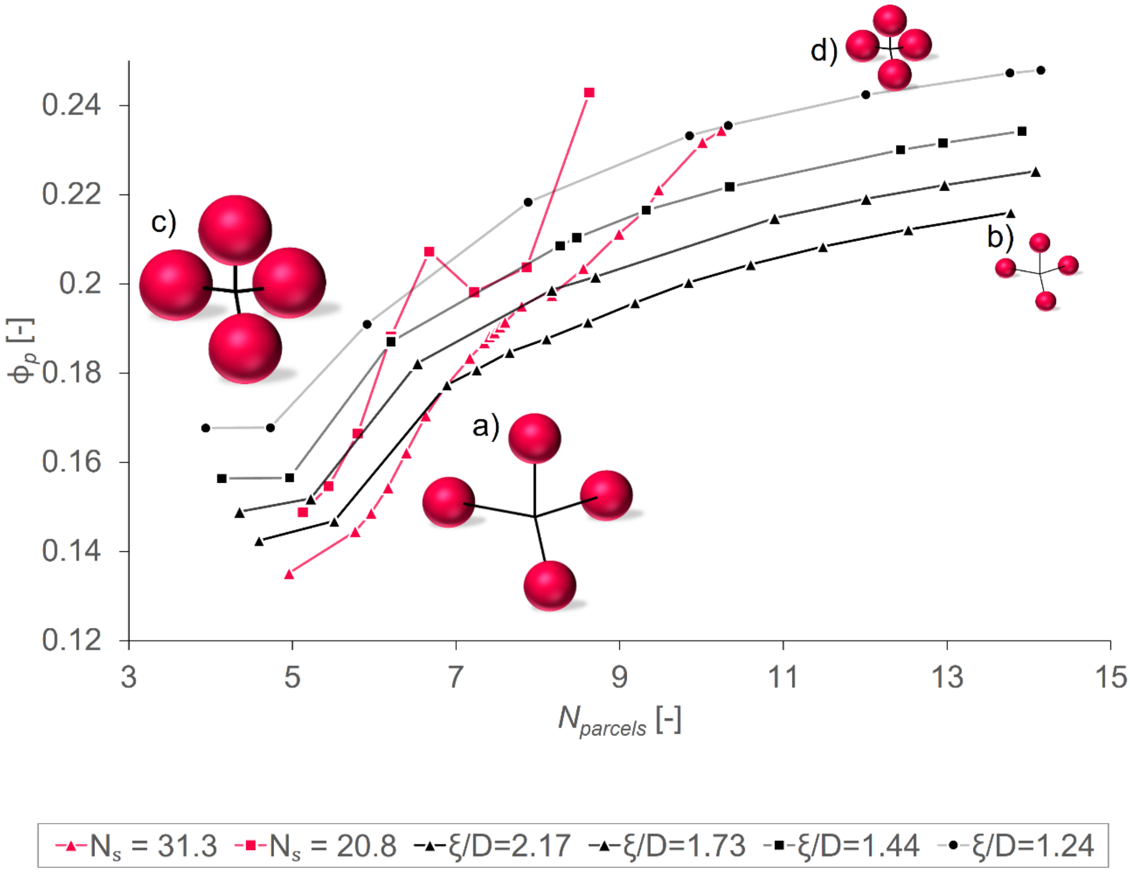

Figure A-4: Mean particle volume fraction over number of parcels for constant D^* ($N_S = 31.3$, $N_S = 20.8$) and constant ξ/D-fractions; confidence intervals are within 1% of $\bar{x}$, and hence omitted; insets show typical tetrapods with $N_S = 31.3$ and large ξ/D for a) $N_{parcel} \approx 7$, b) $N_{parcel} \approx 14$, and small ξ/D for c) $N_{parcel} \approx 4$ and $N_{parcel} \approx 13$.

A.5 Application

Using the methodology described in section 3.5, one can create an intersection plot to find the optimal parameter set for a given resulting distribution of target values (e.g., for the heap fraction). In case two target values are expected, the datasets can be normalized by their respective target values (z) and plotted at z = 1. Doing so, the dimensionality of the 3D-planes reduce to 2D curves. This leads to an easily interpretable resulting intersection of two curves, yielding the optimal parameter combination. Using this information as an input to DEM simulations and comparing the gained results to the given target values, one can validate the accuracy of the used correlations. Exemplarily, the intersection for case 2 shown in Table 2 is illustrated in Figure A-5.

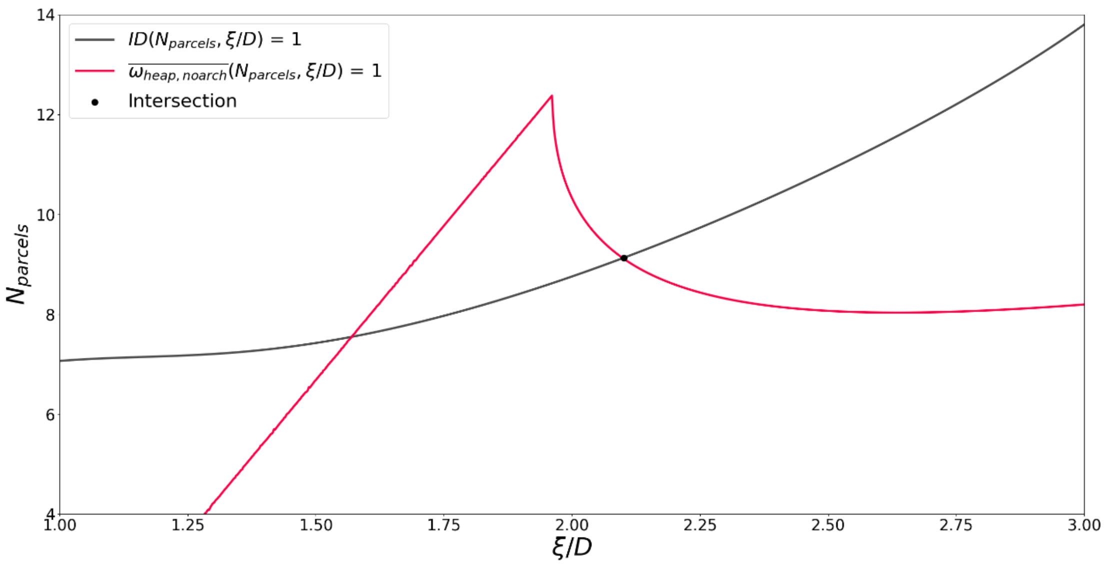

Figure A-5: Intersection plot for normalized (i) $\overline{\omega_{heap,noarch}}$ and (ii) *ID* using target values for case 2 (see Table 2); the intersection points shows the resulting optimal configuration for the given case 2.

A.6 Influence of further particle parameters

To definitively compare the relevance of our findings' impact on the overall simulation uncertainty, we compare the variability observed by us with the one documented by Dahl et al.[2].

Specifically, using the approach established by Roy and Oberkampf [3], Dahl et al. [2] investigated the effects of aleatory (i.e., the distribution of a variable is known) and epistemic (i.e., the distribution is unknown, a uniform distribution is assumed) variables in a DEM simulation. For our purposes, we investigated five different aleatory variables. Adapting the published approach to the setup used in our present work, the lower and upper bounds for the sliding and rolling friction coefficient, as well as the cohesion energy density were set using data from Table 2 presented by Dahl et al.. As the published work only investigated sliding friction, the bounds for rolling friction were proportionally scaled. For k_{sjkr}, an average width of distribution was assumed as this variable was not investigated in the referenced work of Dahl et al.[2] The exact assumptions and values used for said simulations are stated in Table A-1.

For finding each of the five p-boxes (i.e., the lower and upper bound of values for each case), corresponding to the sliding friction coefficient between particle and wall $\mu_{rs,pw}$, between particles $\mu_{rs,pp}$, the rolling friction coefficient between particle and wall $\mu_{rr,pw}$, between particles $\mu_{rr,pp}$, and the cohesion energy density $k_{sjkr}{}^{*}$,respectively, 500 simulations were performed. Each case was run using five different random seeds with 100 simulations sampling from the distributions defined in Table A-1. The resulting p-boxes, as well as each corresponding mean CDF, and the base case mean CDF are shown in Figure A-6**.**

Table A-1: Values used in this work adjusted to a same range as Dahl et al. under the assumption, that they used $\bar{x} \pm 3s \approx 99.7\%$ as upper and lower bounds (Dahl et al. did not state the experimental CDF

used by them); cohesion energy density is assumed to have a distribution with the average width of the frictional variables defined by Dahl et al.[2].

Variable	μ	σ	Variable in Dahl et al. [2](Table 2)
$\mu_{rs,pw}$	0.41	0.11	Particle-wall coeff of friction
$\mu_{rs,pp}$	0.71	0.12	Particle-particle coeff of friction
$\mu_{rr,pw}$	0.80	0.21	N/A
$\mu_{rr,pp}$	0.80	0.14	N/A
$k_{sjkr}{}^{*}\,[J/m^3]$	$3.1{\cdot}10^5$	$6.67{\cdot}10^4$	N/A

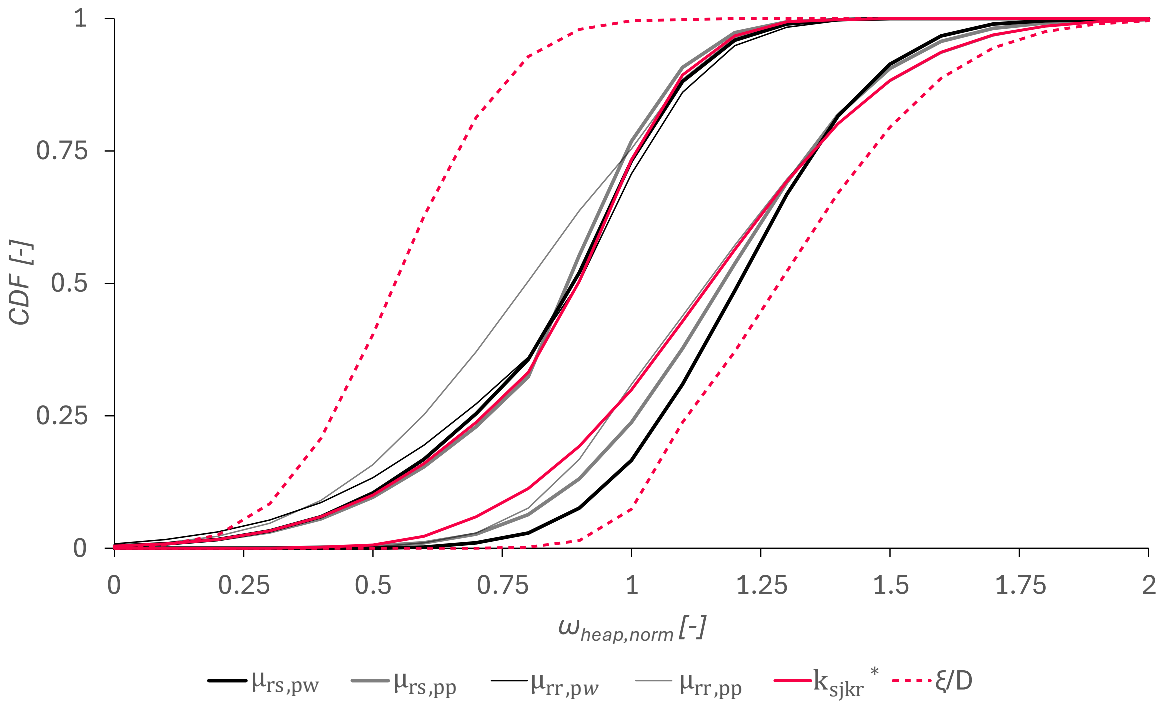

Figure A-6: P-boxes of number-based cumulative density function CDF of the normalized heap mass fraction $\omega_{heap,norm}$ for five different normally distributed random variables of (i) sliding friction coefficient between particle and wall $\mu_{rs,pw}$, (ii) between particles $\mu_{rs,pp}$, (iii) rolling friction coefficient between particle and wall $\mu_{rr,pw}$, (iv) between particles $\mu_{rr,pp}$, and (v) the cohesion energy density $k_{sjkr}{}^{*}$, each using distribution parameters according to Dahl et al.[2] and as shown in Table A-1 (black dashed shaded lines). In addition, the p-box for five different ξ/D-values (compare to Figure 2) are plotted in red dashed lines to compare the width of the p-boxes.

Generally, the width of the distribution is not strongly influenced by the investigated variations of the input variables. Clearly, their impact is limited to shifting $\overline{\omega_{heap,norm}}$. Comparing the p-boxes to the impact of all other previously mentioned factors, such as the size and shape of the parcels, the latter seems to be clearly more important. This becomes apparent, when comparing the size of the p-box of five different ξ/D-values to the five different normally distributed random variables treated in this section,

as shown in Figure A-6. Thus, the important work of Dahl et al. [2] – when applied to our tetrapod approach - suggests that variability caused by particle properties is smaller than that caused by the configuration of tetrapods.

A.7 References

1. Massey, F. J. The Kolmogorov-Smirnov Test for Goodness of Fit. *J Am Stat Assoc* **46**, 68–78 (1951).
2. Dahl, S. R., LaMarche, W. C. Q., Liu, P., Fullmer, W. D. & Hrenya, C. M. Toward reducing uncertainty quantification costs in DEM models of particulate flow: Testing simple, sensitivity-based, forward uncertainty propagation techniques. *Powder Technol* **398**, (2022).
3. Roy, C. J. & Oberkampf, W. L. A comprehensive framework for verification, validation, and uncertainty quantification in scientific computing. *Comput Methods Appl Mech Eng* **200**, 2131–2144 (2011).
